# Peptide cages: bioinspired supramolecular architectures for next-generation applications

**DOI:** 10.1039/d5sc08607h

**Published:** 2026-02-26

**Authors:** Simone Adorinni, Houyang Xu, Jonathan R. Nitschke, Silvia Marchesan

**Affiliations:** a Yusuf Hamied Department of Chemistry, University of Cambridge CB2 1EW Cambridge UK jrn34@cam.ac.uk; b Chemical and Pharmaceutical Sciences Department, University of Trieste 34127 Trieste Italy smarchesan@units.it; c INSTM, University of Trieste 34127 Trieste Italy

## Abstract

This review examines the design and synthesis of peptide-based supramolecular cages, highlighting the versatility and functional diversity achievable by incorporating small peptides as structural components. Inspired by natural supramolecular architectures, synthetic peptide cages offer unique advantages, including tunable chirality, structural predictability, biocompatibility, and ease of functionalisation. The discussion focuses on two principal strategies. The first involves cages in which peptides constitute the primary structural framework, with cage geometry dictated either by intrinsic backbone conformations or by externally imposed directional interactions such as metal coordination. The second covers hybrid systems in which peptides play a functional rather than framework-determining role and are integrated with rigid aromatic or synthetic scaffolds that define the overall architecture. These approaches enable precise control over cage geometry, cavity characteristics, and dynamic behaviour, facilitating applications in biosensing, targeted drug delivery, molecular separation, and environmental remediation. By bridging principles from natural assembly and synthetic supramolecular chemistry, peptide cages represent a powerful platform for developing next-generation functional materials.

## Abstract 2

This review analyses peptide-based supramolecular cages, their design and applications.

## Graphical abstract

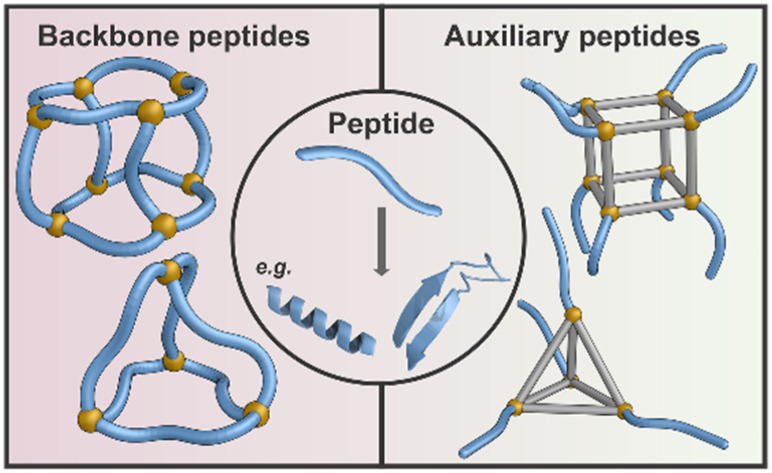

## 1. Introduction

Proteins have evolved to form complex topological structures, spontaneously tying molecular knots,^1^ weaving molecular chainmail,^2^ and creating interlocked architectures of remarkable complexity.^3^ They fold and self-assemble into intricate shapes beyond simple globular forms, including catenanes and interlocked assemblies.^4^ The identification of over 2000 topologically entangled proteins^5^ highlights nature's frequent use of complex molecular architectures, such as the trefoil knots found in methyltransferases and the molecular chainmail structure in HK97 bacteriophage capsids, where cyclic proteins interlock to form catenanes, providing mechanical strength.^2,6^

Additionally, living systems extensively employ protein cages, hollow architectures assembled from multiple protein subunits into defined geometric configurations. Ferritin exemplifies such cages, spontaneously assembling 24 identical subunits into octahedral structures to sequester and regulate potentially harmful metal ions.^7,8^ Similarly, bacterial microcompartments create polyhedral shells from hexagonal protein tiles, spatially isolating reactive metabolic intermediates.^9,10^ Vault particles further illustrate biological complexity, forming large barrel-shaped structures from 78 protein copies that selectively facilitate molecular transport across cellular boundaries.^11,12^ Collectively, these examples underscore how nature assembles proteins into capsular structures to solve biological problems related to molecular organisation and compartmentalisation, beyond the capability of individual protein chains.

Mimicking the biological functions of proteins through more accessible molecular scaffolds has become an appealing strategy due to the synthetic challenges associated with producing full-length proteins. Small peptides, ranging from 2–20 amino acids in length, represent excellent minimalistic motifs for protein-like functions, owing to their numerous advantages, including structural predictability, tunability and synthetic feasibility.^13^ Being essentially homologous to proteins, peptides inherit their non-toxic nature and chirality, making them promising for biological or medical applications.^14^ Rationally designed peptide sequences enable precise control over supramolecular assemblies by leveraging specific amino acid combinations to adopt defined conformations or secondary structures, including α-helices,^15–17^ β-sheets,^18–20^ and coiled coils.^21–23^ This control applies fundamental supramolecular chemistry principles through collective non-covalent interactions, including hydrogen bonding, hydrophobic effects and electrostatic forces, producing adaptive materials that respond dynamically to environmental stimuli while preserving structural integrity.^14,24^ Recent computational advances and machine learning methodologies further enhance the rational engineering of peptide-based systems, enabling targeted assembly into nanostructures with predetermined functionalities.^25^

At the same time, the inherent ability of proteins to form complex capsular structures, coupled with their intrinsic cavities and structural adaptability, has inspired the design of synthetic counterparts.^26,27^ Supramolecular cages are discrete, three-dimensional assemblies formed through cooperative self-assembly of complementary building blocks, featuring well-defined internal cavities ideal for selective guest encapsulation.^28^ Their design requires careful selection of building blocks to promote precise assembly of discrete structures. Notable examples demonstrate how biological assemblies, such as viral capsids and ferritin cages, have directly inspired synthetic cage design.^29,30^ Conformationally adaptable metal–organic cages exemplify this biomimetic approach by dynamically optimising their cavity shapes and volumes, mirroring the induced-fit binding mechanisms observed in proteins.^31–33^ These synthetic assemblies replicate key biological principles while offering controllable mechanical properties and multi-compartmental guest-binding functionalities.^30,34^

Synthetic cages have demonstrated sophisticated capabilities in biomolecular encapsulation. Self-assembled porphyrin-faced cubic cages can sequester and protect long peptides from proteolytic degradation.^35^ Similarly, giant Pd^II^ coordination cages have successfully encapsulated proteins such as ubiquitin, highlighting the synthetic potential to achieve controlled biomolecule protection and functional modulation.^36,37^

Despite these advances in purely artificial supramolecular cage systems, new applications call for enhanced features, including adaptability and stereochemical control. Increased interest is now focused on utilising peptides as structural elements for cage construction rather than merely as guest molecules.^38^ The integration of cage structural design principles with peptide organisation strategies enables researchers to develop peptide cages exhibiting biocompatibility, biodegradability, intrinsic chirality, programmable recognition motifs, and structural flexibility.^39^ Such flexibility confers adaptive cavity behaviour, enabling dynamic responses to guest molecules. The modularity inherent in peptide sequences further facilitates systematic property adjustments through amino acid modifications.

**Fig. 1:**
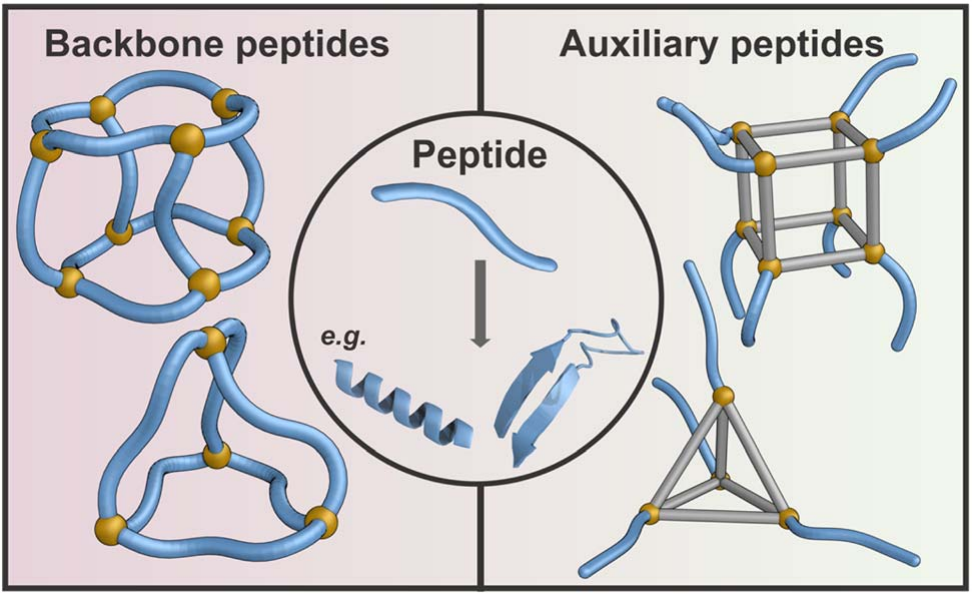
The central circular panel illustrates a generic short peptide (2–20 amino acids) as a blue tubular strand, capable of adopting diverse secondary structures that include an α-helix and a β-sheet, which can serve as a modular subunit for cage design. Two distinct strategies for its incorporation are highlighted. On the left, cages with peptides as backbones are constructed from peptide-based building blocks, where self-assembly is directed by the conformational preferences and interaction motifs encoded in the sequence. On the right, cages with peptides as auxiliary units integrate short peptides with rigid aromatic scaffolds, where the peptide contributes additional recognition or functional features outside the core. All structures shown are schematic representations.

This review focuses on supramolecular cages incorporating peptides, with particular emphasis on peptide involvement in the framework, backbone conformation, and bonding strategy, which together exert structural control within the assembly. Two principal design approaches are discussed (Fig. 1). The first approach, which is the focus of Section 2, centres on peptides that incorporate key structural motifs or predominantly constitute the framework of resulting structures, enabling the backbone to either adopt defined conformations reminiscent of protein secondary structure elements, or to remain conformationally flexible as other driving forces provide directional control. In these systems, the peptide backbone functions as an integral architectural component of the cage, actively contributing to geometry, connectivity, and internal organisation. Metal coordination is frequently employed as a strong and directional interaction to stabilise and reinforce peptide-encoded geometries, rather than to impose cage structure independently.

The second approach, described in Section 3, encompasses systems mostly based on amino acids or short peptides, which play a framework-decorating role and do not encode cage geometry through backbone conformation. These peptides are linked to rigid, typically aromatic scaffolds that define the overall cage architecture, playing primarily a functional role.

These systems exploit the chemical diversity and reactivity of amino acid side chains to introduce recognition elements, responsiveness, or tuneable chemical properties. As a result, this class includes cages assembled through a range of strategies, including metal ligand coordination, covalent and dynamic covalent chemistry, and hydrogen bonding interactions.

We recognise that overlap exists between the concepts of cages incorporating ‘framework’ and ‘peripheral’ peptides. Several cases are highlighted below where peptides simultaneously contribute to both structural organisation and functional modulation.

Finally, Section 4 discusses the practical applications of peptide based supramolecular cages, illustrating how these distinct design strategies translate into performance in biosensing, drug delivery, molecular separation, and environmental remediation, while also highlighting current limitations related to stability, scalability, and functional robustness.

## 2. Cages with peptides as backbones

A key advantage of employing peptides as building blocks for self-assembly is their versatility and, to some extent, predictability of folding. To overcome the entropic penalty inherent in self-assembly, conformational preorganisation of ligands is often necessary.^40,41^ Consequently, rigid ligands have been employed in studies of supramolecular assemblies.^41^ When low-symmetry and structurally complex architectures are required to bind more intricate target molecules, peptide-based backbones in supramolecular assemblies have caught the attention of researchers. The use of peptides effectively employs protein-like folding characteristics for the formation of small-molecule assemblies. A judicious choice of amino acids, according to the specific ranges of *ϕ* and *ψ* torsion angles, enables the rational design of geometries between the building blocks, such as angles between chelating sites,^42^ and the controlled twisting of the peptide strands forming the ligands. Amino acidic side chains, both natural and unnatural, also provide additional connectivity options. These side chains may provide sites for molecular recognition,^43^ form secondary structures integral to the assembled architecture,^44^ or directly engage in metal coordination.^45–49^ In this section, we discuss representative examples highlighting the use of peptides in constructing self-assembled capsular structures.

### 2.1 Metal-directed peptide cages

**Fig. 2:**
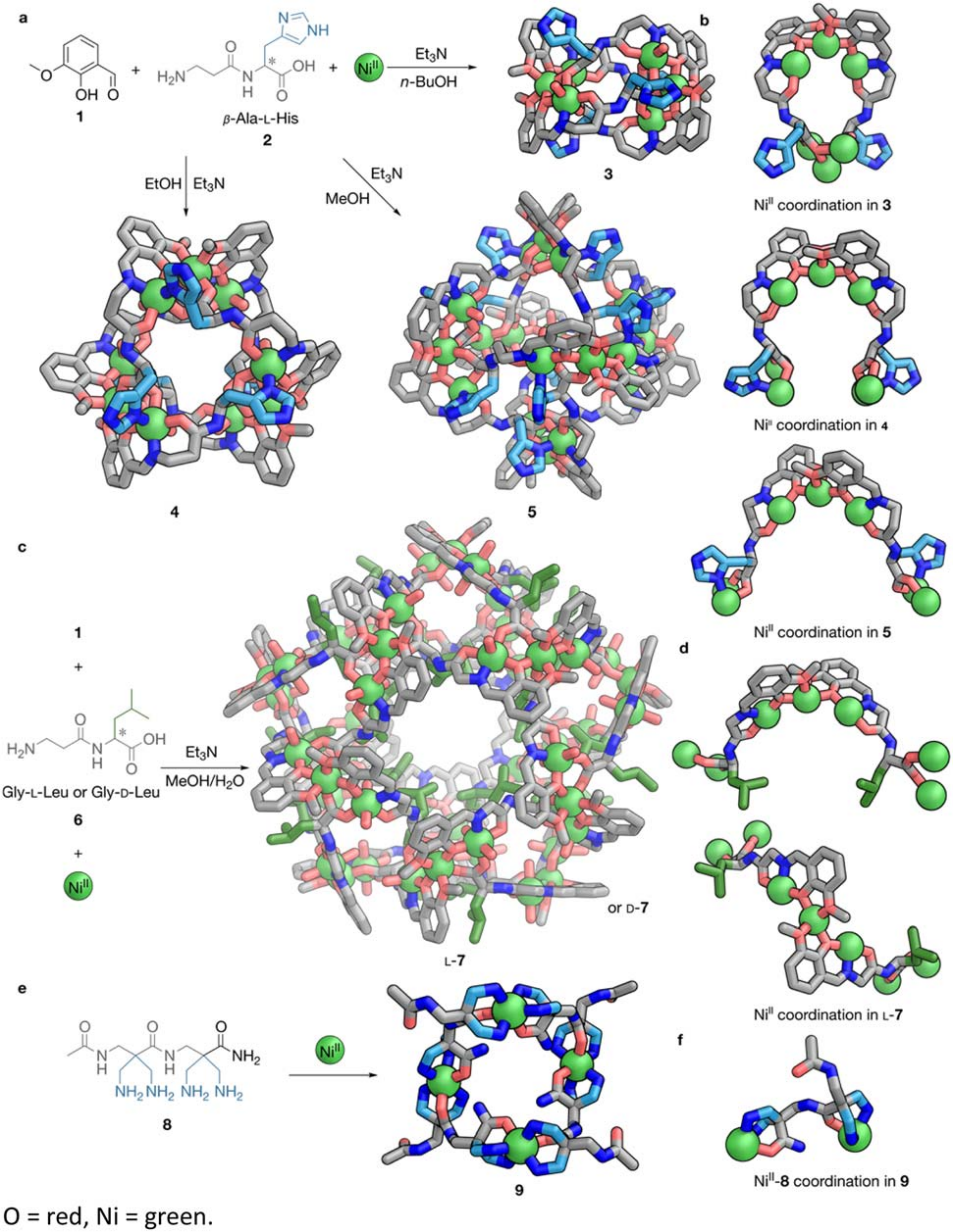
Peptide-backbone cages and macrocycles from peptides folded under the direction of metal coordination. (a) The subcomponent self-assembly of *o*-vanillin (1), dipeptide ligand 2 and Ni^II^ generates [Ni^II^_6_L_4_]^4+^ helicate 3 when *n*-BuOH is used as solvent. Changing the solvent to EtOH or MeOH generates [Ni^II^_9_L_6_]^6+^ triangular capsule 4 and [Ni^II^_18_L_12_]^12+^ octahedron 5, respectively. The structures of these cages are shown. (b) Visual comparison of the coordination modes between Ni^II^ and the ligand generated from imine condensation of 1 and 2 in structures 3–5; note the increase of the opening angle between each pair of ligands. (c) The subcomponent self-assembly of 1, dipeptide ligand 6 and Ni^II^ generates [Ni^II^_45_L_30_] helicate 7 when a mixed MeOH/H_2_O solvent is used. The chirality of 7 depends on the handedness of the Val-residue of 6. The structure of L-7 is visualised. (d) Visualisation of coordination between Ni^II^ and the imine ligand formed by 1 and 6 in cage L-7. (e) The self-assembly of amine ligand 8 and Ni^II^ generates [Ni^II^_4_L_4_]^8+^ macrocycle 9. (f) Visualisation of coordination between Ni^II^ and 8 in macrocycle 9. All structures are single-crystal X-ray diffraction (SCXRD) structures. Colour codes: C = light blue (basic side chains), green (non-polar side chains) or grey (other moieties), N = blue, O = red, Ni = green.

When peptides are too short or flexible to adopt defined conformations, non-covalent forces can guide their structural organisation. This process is facilitated by adoption of potentially preferred geometries^50^ and solvophobic effects^51^ that further restrict conformations. A notable case is the [Ni^II^_3_]–dipeptide cluster motif developed by Kong *et al.*^51,52^ Imine capping with *o*-vanillin (1) of the β-Ala–l-His (2) N-terminus gives a multidentate ligand: the aldehyde residue, imine nitrogen and β-Ala amide bind two Ni^II^ ions, while the C-terminal carboxylate and His side chain can potentially chelate two additional metals (Fig. 2a). Three Ni^II^ centres then assemble two ligand strands into a head-to-head N-terminus junction holding a Ni^II^_3_ cluster together, and the cluster's open coordination sites engage the C-termini of neighbouring strands, forming a polyhedral network. Kong and co-workers discovered that self-assembly in the presence of base in *n*-butanol, ethanol, and methanol led to the formation of [Ni_6_L_4_]^4+^ helicate 3, [Ni_9_L_6_]^6+^ triangle 4, and [Ni_18_L_12_]^12+^ octahedron 5, respectively, with increasing opening angles between edges and increasing *φ* (−172.36° to −131.90°) and *ψ* (−173.65° to −158.20°) torsion angles of the peptides (Fig. 2a–d). This range of linkage geometries demonstrates that the peptides retain a degree of flexibility even after coordination to the Ni^II^_3_ cluster. Increased flexibility, combined with solvophobic effects, can thus lead to large capsular structures. Condensation of *o*-vanillin and another dipeptide, Gly–l-Leu (L-6) or Gly–d-Leu (D-6) results in a more compact, flexible and hydrophobic Ni^II^_3_ cluster motif, which self-assembles into a large [Ni_45_L_30_] cage 7, as shown in Fig. 2c.^51^ The greater flexibility of Gly allowed the coordinated peptide to adopt multiple conformations within the same structure (Fig. 2d), with *φ* ranging from −87.3° to −136.56° and *Ψ* from −175.94° to 84.80°. The isobutyl side chain of Leu tends to point inward due to solvophobic effects in the polar MeOH/H_2_O solvent used for self-assembly, and side chain bulk was identified as a driving force for the formation of the large capsular structure.

Vázquez *et al.* reported peptide-based helicate systems where designed sequences encode structural information, with metal coordination reinforcing the resulting geometry. Oligocationic peptide ligands containing six bipyridine residues fold into chiral three-stranded helicates in the presence of Fe^II^ or Co^II^ ions. Heterochiral β-turn sequences with l-Arg–l-Pro–d-Arg encode stereoselective folding, directing assembly into ΛΛ- or ΔΔ-helicates under thermodynamic control. Two isomers with identical stereochemistry but different bipyridine connectivity were identified through NMR spectroscopy. A Cu^II^ variant of this system was subsequently developed, demonstrating the structural versatility of this peptide scaffold.^53,54^

Another motif that demonstrates non-covalent templation of peptide folding in self-assembly is Miyake *et al.*'s artificial β-oligopeptides bearing tridentate propanediamine side chains. These peptides cyclise *via* Ni^II^-mediated self-assembly into macrocyclic clusters, with the first report being the self-assembly of β-dipeptide 8 into [Ni^II^_4_L_4_]^8+^ macrocycle 9, as shown in Fig. 2e. In this structure, C- and N-terminal tridentate donors each bind two Ni^II^ ions (Fig. 2f), engendering a macrocyclic structure with an internal cavity for guest inclusion.^45,55^

### 2.2 Metallomacrocycles and interlocked topologies from interplay of coordination and peptide folding

**Fig. 3:**
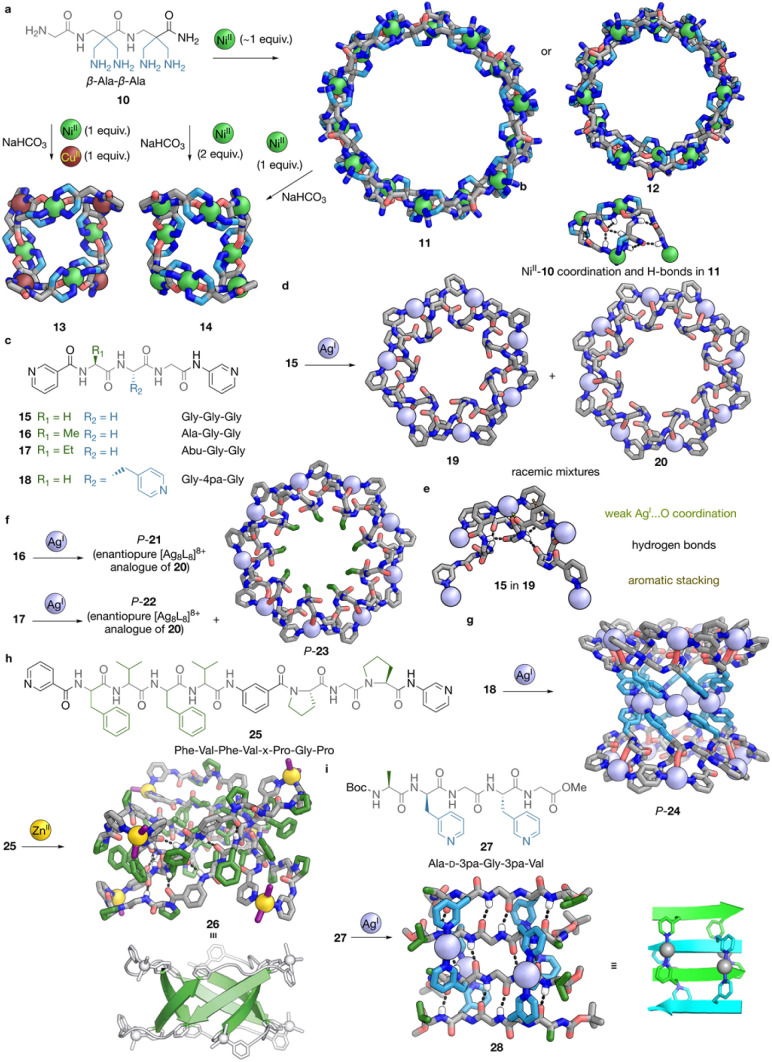
Cage and interlocked structures with peptide backbones, whose formation is directed by peptide folding and secondary interactions with metal coordination. (a) The structure of amine ligand 10 and its sequence, and its various self-assembly reactions. The self-assembly of 10 and Ni^II^ yields [Ni^II^_14_L_14_]^28+^ macrocycle 11 or [Ni^II^_12_L_12_]^24+^ macrocycle 12 under different crystallisation conditions. Using 2 equiv. of Ni^II^ and NaHCO_3_, or adding 1 equiv. of Ni^II^ and NaHCO_3_ to 11 or 12, affords [Ni^II^_8_L_8_]^16+^ macrocycle 14. Using 1 equiv. of Ni^II^, 1 equiv. of Cu^II^ and NaHCO_3_ generates [Ni^II^_4_Cu^II^_4_L_8_]^16+^ macrocycle 13 instead. (b) Visualisation of coordination between Ni^II^ and ligand 10 and hydrogen bonds in macrocycle 11. (c) The structures of bis-pyridyl ligands 15–18, and their sequences. (d) The self-assembly of ligand 15 and Ag^I^ to give torus knots 19 ([Ag^I^_7_L_7_]^7+^) and 20 ([Ag^I^_8_L_8_]^8+^) as racemic mixtures. (e) Visualisation of coordination between Ag^I^ and ligand 15 and secondary interactions in torus knot 19. (f) The self-assembly of ligand 16 and Ag^I^ forms P-21, an enantiopure [Ag^I^_8_L_8_]^8+^ analogue of 20, while the self-assembly of ligand 17 and Ag^I^ forms a mixture of P-22, an enantiopure [Ag^I^_8_L_8_]^8+^ analogue of 20, and enantiopure [Ag^I^_9_L_9_]^9+^ torus knot P-23. (g) The self-assembly of ligand 18 and Ag^I^ forms P-24, an enantiopure [Ag^I^_21_L_14_]^21+^ barrel. (h) The structure of bis-pyridyl ligand 25 with its sequence shown and its self-assembly with Zn^II^ to form Zn^II^_6_L_6_I_12_ β-barrel 26, with hydrogen bonds visualised on its structure, and a minimal visualisation of its secondary structure adapted from ref. 55, copyright © 2018 American Chemical Society. (i) The structure of bis-pyridyl ligand 27 and its self-assembly with Ag^I^ to form β-sheet [2]catenane 28, with hydrogen bonds visualised on its structure, and a visualisation of its secondary structure, as adapted from ref. 58, copyright © 2024 the author(s). *Angew. Chem., Int. Ed.* published by Wiley-VCH GmbH. All structures shown are SCXRD structures. Colour codes: C = light blue (basic side chains), green (non-polar side chains) or grey (other moieties), N = blue, O = red, Ni = green, Cu = brick red, Zn = yellow, Ag = silver, I = purple, hydrogen bonds = black, aromatic stacking = brown, weak AgI⋯O coordination = lime.

In the formation of peptide-backboned self-assembled structures, metal coordination can not only independently guide peptide conformation, but may also act synergistically with intra- or inter-chain interactions, enhancing overall structural control. This cooperativity extended Miyake's motif,^45^ providing access to larger macrocyclic structures.^46^ Starting from the first [Ni^II^_4_L_4_]^8+^ macrocycle 9, changing the acetyl at the N-terminus into a Gly residue provides another metal binding site for the resulting β-tripeptide ligand 10. With Ni^II^, a range of larger flexible macrocyclic structures can be formed, as shown in Fig. 3a. Crystallisation under different conditions afforded [Ni^II^_14_L_14_]^28+^ macrocycle 11 and [Ni^II^_12_L_12_]^24+^ macrocycle 12. In both structures, intra-chain H-bonding and metal binding curl the peptides into zig–zag shapes, organising the repeating units of bent peptides and Ni^II^ centres into macrocyclic structures (Fig. 3b). The amide groups in 10 can be deprotonated under basic conditions to participate in coordination. Self-assembly with a 1 : 1 mixture of Ni^II^ and Cu^II^ cations results in the formation of [Ni^II^_4_Cu^II^_4_L_8_]^16+^ macrocycle 13, where Cu^II^ adopts square planar tetra-coordination, with amides and β-alanines as donors. The addition of excess Ni^II^ can also convert 11 to favour tetra-coordination instead of 6-coordination for part of the Ni^II^, resulting in the formation of [Ni^II^_8_L_8_]^16+^ macrocycle 14, a homometallic analogue of 13.^56^

Enhanced inter-chain interactions can also lead to the formation of interlocked structures. As reported by the Fujita group, [Ag_7_L_7_]^7+^ knot 19 and [Ag_8_L_16_]^8+^ link 20 self-assemble from Ag^I^ and an extremely flexible Gly_3_ peptide 15, with pyridyl functionalisations at both termini for Ag^I^ coordination, as shown in Fig. 3c and d.^57^Fig. 3e shows the non-covalent interactions identified as the driving forces for these interlocked structures to form, including inter-chain H-bonding, aromatic stacking between the pyridyls, as well as C=O⋯Ag^I^ coordination. These interactions cooperatively organise the flexible triglycine peptide into a single conformation in self-assembly, where the three glycines show distinct *φ* and *Ψ* torsion angles (Gly2: *φ* ∼ −60°, *Ψ* ∼ 180° *vs.* Gly_3_: *φ* ∼ 60°, *Ψ* ∼ −180°).^48^ Gly2 could be replaced with Ala, which can adopt similar torsion angles. The chirality of Ala biases the helicity of the originally-racemic links into an enantiopure form, as shown by the formation of P-21 from the self-assembly of 16 and Ag^I^ (Fig. 3f).^48^

In order to access knots and link structures with more crossings, Sawada and Fujita *et al.* sought to introduce strain to the Gly1 or Ala1 residue (in 15 and 16, respectively) by mutation to amino acids with bulkier inward-pointing side chains. As shown in Fig. 3f, the use of l-2-aminobutyric acid (Abu) in ligand 17 extended the range of self-assembled structures accessible, generating a mixture of 22, an 8-crossing link analogue of 20, and 9-crossing [Ag_9_L_9_]^9+^ knot 23. The use of norvaline (Nva) or norleucine (Nle) gave rise to even larger [Ag_10_L_10_]^10+^ links and extended supercoil structures.^58^ Furthermore, as shown in Fig. 3g, Gly2 can also be replaced by chiral 4-pyridylalanine (4pa) to generate ligand 18, which can access similar torsion angles to form the [Ag_7_L_14_]^7+^ torus link structure, while the additional pyridyls can coordinate to 7 extra Ag^I^ vertices in a linear manner, to form [Ag_21_L_14_]^21+^ peptide nanotube 24.^48^

The promotion of inter-chain interactions by metal coordination can be further exploited to create secondary structures similar to those of proteins. In 2018, Sawada, Fujita *et al.* reported synthetic [Zn_6_^II^L_6_]^12+^ β-barrel 26, in which secondary structure folding was facilitated by metal coordination.^44^ As illustrated in Fig. 3h, 26 self-assembled from peptidic chain 25, comprising two Phe–Val–Phe–Val and Pro–Gly–Pro peptidic chains, linked together by a 1,3-phenylene linker (x), functionalized with 3-pyridyl at both termini. Self-assembly with Zn^II^ forms a [Zn^II^L_2_]^4+^ macrocycle, with two coordinating halide counterions per vertex. The Phe–Val–Phe–Val sequence has an intrinsic tendency to form a β-barrel, which was enhanced by the geometry of Zn^II^ coordination. The flexible Gly adopts an extended conformation (*φ* ∼ −100°, *Ψ* ∼ −170°), forming an S-shaped strand for the Pro–Gly–Pro moiety with more robust, twisted Pro residues (*φ* ∼ −110 to −70°, *Ψ* ∼ 120 to 180°), complementing the extended Phe–Val–Phe–Val (*φ* ∼ −150 to −70°, *Ψ* ∼ 120 to 150°) to form the macrocyclic structure, thereby allowing for the recognition among the Phe–Val–Phe–Val side chains to generate the β-barrel.

Metal-directed folding also provided a pathway to access other secondary structures, including double-stranded β-helices (ds-β-helices)^59^ and β-sheets.^47^ By using pentapeptide 27 with Boc protecting groups at the N-termini and methyl esters at the C-termini, featuring 3-pyridylalanine (3pa), with the sequence (Ala–d-3pa–Gly–3pa–Val), self-assembly with Ag^I^ results in the formation of [Ag_4_L_4_]^4+^ [2]catenane 28 (Fig. 3i).^47^ The coordination of 3pa with Ag^I^ generates macrocyclic components with the two peptide strands separated to a sufficient degree to accommodate a strand from another macrocycle, and the β-sheet recognition and metal coordination mutually result in the catenation of 28.

**Fig. 4:**
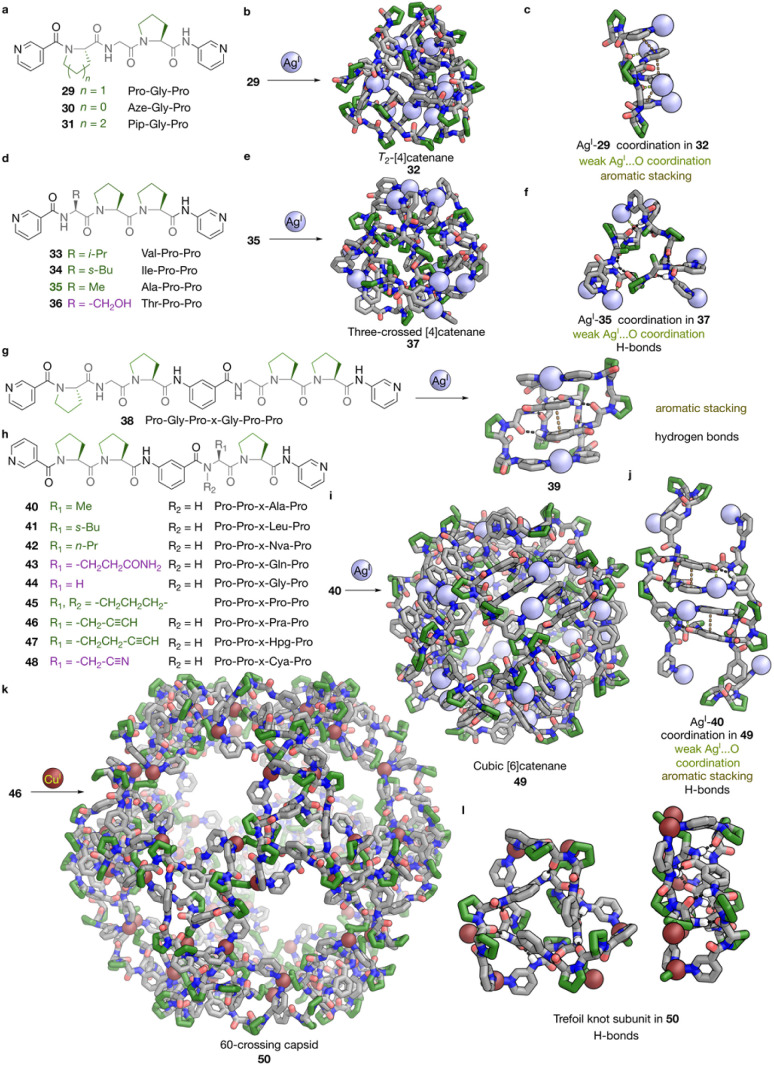
Peptide-backboned interlocked cage structures from the interplay of peptide folding with metal coordination. (a) The structures of bis-pyridyl ligands 29–31 and their sequences. (b) The self-assembly of ligand 29 and Ag^I^ to T_2_-type tetrahedral [Ag^I^_12_L_12_]^12+^ [4]catenane 32. (c) Visualisation of coordination between Ag^I^ and ligand 29, and secondary interactions in [4]catenane 32. (d) The structures of bis-pyridyl ligands 33–36, and their sequences. (e) The self-assembly of ligand 35 and Ag^I^ to three-crossed type [Ag^I^_12_L_12_]^12+^ [4]catenane 37. (f) Visualisation of coordination between Ag^I^ and ligand 35 and secondary interactions in [4]catenane 37. (g) The structure of bis-pyridyl ligand 38 and its self-assembly with Ag^I^ to form [2]catenane 39, with secondary interactions visualised on its structure. (h) The structures of bis-pyridyl ligands 40–48, and their sequences. (i) The self-assembly of ligand 40 and Ag^I^ to cubic [Ag^I^_24_L_24_]^24+^ [6]catenane 49. (j) Visualisation of coordination between Ag^I^ and ligand 40 and secondary interactions in [6]catenane 49. (k) The self-assembly of ligand 46 and Cu^I^ to give icosahedral 60-crossing [Cu^I^_60_L_60_]^60+^ capsid 50. (l) Visualisation of a trefoil knot structural unit in 50, with hydrogen bonds visualised on its structure. All structures shown are SCXRD structures. Colour codes: C = green (non-polar side chains) or grey (other moieties), N = blue, O = red, Cu = brick red, Ag = silver, hydrogen bonds = black, aromatic stacking = brown, weak AgI⋯O coordination = lime.

Fujita and Sawada *et al.* have thus unveiled the entry to a series of metal-peptide interwoven structures where folding is key to structure formation.^49,60–64^ In 2016, they reported the self-assembly of Ag^I^ with a peptidic bis-pyridyl ligand 29 (Fig. 4a), with a sequence of Pro–Gly–Pro.^60^ The cooperative effect of the robust Pro units and Ag^I^ coordination led to an Ω-loop conformation, in which the two Pro exhibit typical torsion angles (*φ* ∼ −65°, *Ψ* ∼ 150 or −30°) similar to the α-helix region, while Gly, in contrast to the extended configuration adopted in 28, showed a harsher twist (*φ* ∼ −120°, *Ψ* ∼ 180°). This conformation eventually leads to the assembly of T_2_-type [Ag^I^_12_L_12_]^12+^ [4]catenane 32 with 12 crossings (Fig. 4b and c). Assembly with ligand 30, in which Gly_1_ was replaced with azetidine-2-carboxylic acid (Aze), a more robust, four-membered ring version of Pro, resulted in the formation of another T_2_-type [Ag^I^_12_L_12_]^12+^ [4]catenane analogue of 30. The use of 31, using six-membered ring piperidine-2-carboxylic acid (Pip), did not form the same structure, which indicates the importance of the rigidity of the peptide in the folding and assembly process, allowing positioning of secondary interactions at the required locations.

Changing this sequence to Gly–Pro–Pro promotes the formation of a near-linear PP_II_-helix conformation instead of an Ω-loop, leading to the formation of an infinite network.^65^ Mutation of the Gly to ligands 33 or 34 (shown in Fig. 4d) with bulky Val or Ile regenerates the Ω-loop, forming [Ag^I^_12_L_12_]^12+^ [4]catenanes with the same topology as 32. However, when smaller Thr or Ala is used for a Ala–Pro–Pro (ligand 35) or Thr–Pro–Pro (ligand 36) sequence, a different type of three-crossed [Ag^I^_12_L_12_]^12+^ [4]_12_ catenane structure was found to self-assemble, with the Ala-containing 37 shown in Fig. 4e and f, featuring PP_II_-helices.^62^ For 34 (Ile–Pro–Pro), 35 (Ala–Pro–Pro) or 36 (Thr–Pro–Pro) (Fig. 4d), despite showing a similar range of torsion angles (*φ* ∼ −120 to −60°, *Ψ* ∼ 90–180°), the peptide bond between the two Pro is *cis* (*ω* = 0°) in 34, which formed the PP_I_ twist required for a similar Ω-loop as in 32, while this peptide bond is *trans* (*ω* = 180°) in the latter two, which retain the PP_II_-helix structure. These PP_II_-helix containing peptides thus form macrocyclic components that interweave in a different way.

Tethering of two of these peptides together with a 1,3-phenylene linker provides access to more types of interlocked structures.^61,63^ For example, dipyridyl ligand 38 (Fig. 4g), with a Pro–Gly–Pro–x–Gly–Pro–Pro sequence, curls upon binding Ag^I^, thus forming an Ω-loop of the Pro–Gly–Pro chain, while the other half of the ligand complements it to generate a macrocycle. Two of these macrocycles were found to interlock to generate [Ag^I^_2_L_4_]^12+^ [2]catenane 39*via* aromatic stacking, with the two Pro–Gly–Pro and Gly–Pro–Pro tripeptide stands showing similar conformations as in their corresponding homomeric structures.^61^ Compound 40, a shorter tethered bis-dipeptidic ligand, with a Pro–Pro–x–Ala–Pro sequence, was found to form a [Ag^I^_24_L_24_]^24+^ cubic [6]catenane 49 (Fig. 4h and i), which contains six macrocyclic peptide tetramers.^63^ Each peptide chain forms an S-shaped loop with a ∼90° turn, with each residue showing a similar degree of PP_II_-helix-like twist (*φ* ∼ −60°, *Ψ* ∼ 130–180°) As shown in Fig. 4j, each cube vertex is stabilised by six sets of inter-chain hydrogen bonds across three ligand strands, while on each edge, four sets of hydrogen bonds were found among the amides from two peptide chains. The cubic link structure of 49 can tolerate mutations at the Ala residue to ligands 41 and 42 containing bulky Leu or Nva,^49^ or polar functionality, such as 43 with Gln, but 44 with the overly flexible Gly or 45 with rigid Pro did not result in the formation of any discrete structure.

More drastic alternation in the formed structures happens when the Ala in the Pro–Pro–x–Ala–Pro sequence is replaced with the coordinating propargylglycine (Pra) of 46, which was found to assemble with Cu^I^ to form gigantic Cu_60_L_60_ dodecahedral link 50 (Fig. 4k).^49^ The Pra residue was essential for the formation of 50 in the sense of both peptide folding and metal coordination. It has the correct geometry to serve as an additional donor to the Cu^I^ cations, and it also closes the ligand strands to form the trefoil knot subunits shown in Fig. 4l, which was considered crucial to the formation of 50. The triangular edges of these knots join together to form the pentagonal windows of the dodecahedral framework, instead of the square windows for the cubic link, with only Pra replacing the Ala in the peptide sequence. This result may be explained by Ramachandran plots of 43 and 45, which also revealed that instead of adopting a typical helical twisted conformation in a PP_II_-helix, the degree of twisting (*φ* ∼ −90°, *Ψ* ∼ 110°) of the Pra residue is more open, between a PP_II_-turn and an extended (β-sheet-like) conformation. This difference may drive the formation of pentagonal instead of square windows. Elongation of the Pra in 46 by one β-methylene to l-homopropargylglycine (Hpg) in 47, or changing the propargyl to the non-coordinating β-cyano-l-alanine (Cya) in 48, led to the formation of non-interlocked structures.

### 2.3 Pre-organised oligoproline metallacages

**Fig. 5:**
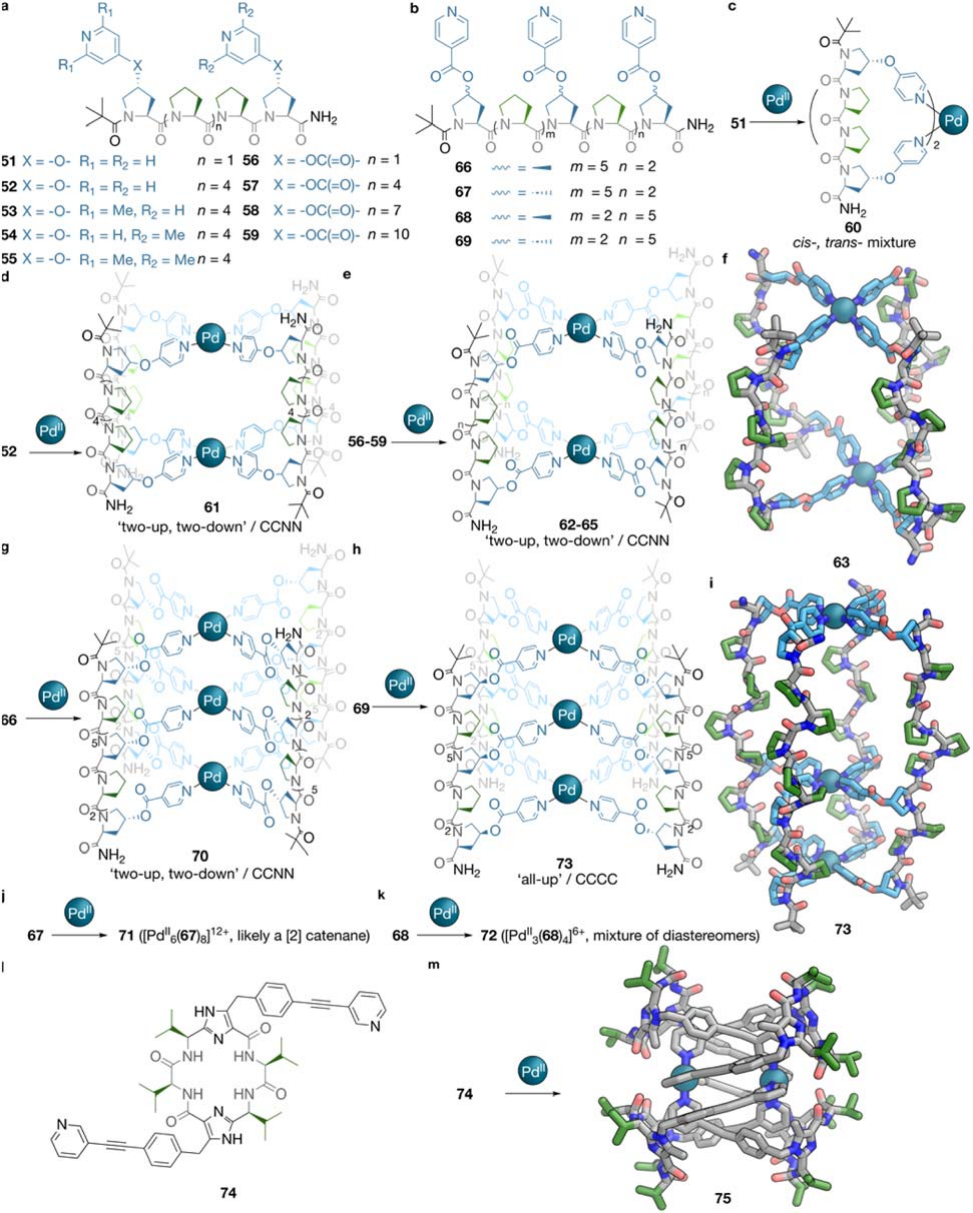
Peptide-backboned cages from robust, highly preorganised peptides. (a) The structures of bis-pyridyl oligoproline ligands 51–59. (b) The structures of tris-pyridyl oligoproline ligands 66–69. (c) The self-assembly of ligand 51 and Pd^II^ to [Pd^II^L_2_]^2+^ mononuclear complex 60 as a mixture of *cis*- and *trans*-stereoisomers. (d) The self-assembly of ligand 52 and Pd^II^ to give [Pd^II^_2_L_4_]^4+^ cage 61 as a ‘two-up, two-down’ or CCNN stereoisomer, considering the orientations of the peptide chains. (e) The self-assembly of ligand 56–59 and Pd^II^ to give [Pd^II^_2_L_4_]^4+^ cages 62–65, respectively. 62–65 are prepared as the ‘two-up, two-down’ or CCNN stereoisomer, regarding the orientations of peptide chains. (f) A model of cage 63. (g) The self-assembly of ligand 66 and Pd^II^ to give [Pd^II^_3_L_4_]^6+^ cage 70 as a ‘two-up, two-down’ or CCNN stereoisomer, regarding the orientations of the peptide chains. (h) The self-assembly of ligand 69 and Pd^II^ to give cage 73 as a ‘all-up’ or CCCC stereoisomer, regarding the orientations of peptide chains. (i) A model of cage 73. (j) The self-assembly of ligand 67 and Pd^II^ to give [Pd^II^_6_L_8_]^12+^ structure 71, inferred to be a [2]catenane. (k) The self-assembly of ligand 68 and Pd^II^ to give [Pd^II^_3_L_4_]^6+^ structure 72, as a mixture of all possible diastereomers, considering the orientations of peptide chains. (l) The structure of bis-pyridyl ligand 74, with a cyclic recognition motif containing four Val-residues in its centre. (m) The self-assembly of ligand 74 and Pd^II^ to give [2]catenane cage 75. Colour codes for schematic drawings: main chains = grey, basic side chains = blue, neutral, non-polar side chains = green, termini and other moieties = black. Colour codes for rendered structures: C = light blue (basic side chains), green (non-polar side chains) or grey (other moieties), N = blue, O = red, Pd = teal.

As described above, metal-directed folding and self-assembly proved to be an efficient approach to allow access to diverse interlocked architectures. Another route to well-defined self-assembled structures consists of using peptides with strong conformational tendencies, and their bridging with non-covalent interactions. This principle is highlighted by recent work on oligoproline metallacages. McTernan *et al.* first demonstrated this approach, with Palma *et al.* subsequently reporting related designs.^66–68^Fig. 5a and b shows the design of the oligo(l-proline) ligands reported by these groups. As it takes approximately three residues for a full 360° turn to occur in a PP_II_ secondary structure, these groups designed ligands that include 4, 7, 10, and 13 residues. Featuring similar designs, all these ligands have the N-termini capped with a pivalic amide structure and C-termini functionalised as amides, with *trans*-hydroxyproline (Hyp) replacing Pro residues at both ends. As shown in Fig. 5a, Palma *et al.* functionalised the hydroxy groups with bare or dimethyl substituted pyridyls, obtaining ligands 51 and 52–55,^67^ whereas McTernan *et al.* prepared ligands 56–59.^66^ The self-assembly of 51 with Pd^II^ yielded a *cis*–*trans* mixture of [Pd^II^L_2_]^2+^ mononuclear complex 60 (Fig. 5c), with stereoisomerism generated by the orientation of the oligoproline strands. The same stereochemistry resulted in four potential isomers (‘all-up’ or CCCC, ‘two-up, two-down’ or CCNN, ‘one up, three down’ or CNNN, and ‘one down, three up’ or CCCN) for the self-assembly of 52 and 56–59. Each one generated lantern-shaped [Pd^II^_2_L_4_]^4+^61 and 62–65, respectively (Fig. 5d and e), with a model of 63 shown in Fig. 5f. Interestingly, 61 was found to adopt CCCC stereochemistry for the peptidic orientations, while 62–65, despite having distinct numbers of turns, all form CCNN diastereomers. Mono-methylated ligands 53 and 54 were observed to generate complex mixtures upon treatment with Pd^II^, while no Pd^II^ complexation to the bulkier lutidine-based 55 was found to take place at all.

Proceeding from the structure of bis-pyridyl ligand 58 (10 Pro residues), McTernan *et al.* then added one extra 4-pyridyl functionalisation to either the Pro_7_ or Pro_3_ as an extra donor site, obtaining ligands 66–69.^68^ As shown in Fig. 5b, 66 and 67, and 68 and 69, are prepared as pairs of diastereomers: the handedness of all β-carbons is either kept as *R*-, as in 51, 52–55 and 56–59, or inverted to *S*-. Four distinct scenarios were observed for these two pairs of stereoisomers. The self-assembly of 66, 68 and 69 yielded [Pd^II^_3_L_4_]^6+^ structures 70, 72 and 73, respectively, with distinct stereochemistries. 70 was found to maintain the CCNN stereochemistry observed for 62–65 (Fig. 5g), while 73 was found to exhibit the CCCC configuration, as shown in Fig. 5h and i. In contrast, 72 was found to assemble as a complementary mixture, including all four potential isomers (Fig. 5k). The stoichiometry of 71, the self-assembled product from 67 and Pd^II^, was identified as [Pd^II^_6_L_8_]^12+^ (Fig. 5j), which was inferred by McTernan *et al.* to be a [2]catenane structure formed by two [Pd^II^_3_L_4_]^6+^ cages. The stereochemistry of these cages could not be determined due to the broad NMR spectra.

Peptide conformations can also be locked covalently before assembly. A 2020 report by Clever *et al.* featured this approach to create a recognition motif for the synthesis of interlocked structures.^43^Fig. 5l shows the C-shaped peptidic ligand 74 employed in this study, featuring two Val–Val strands, brought together in a head-to-tail manner with a methylimidazole ring, functionalised with pyridyl ligating units. As shown in Fig. 5m, its self-assembly with Pd^II^ affords [Pd_2_L_4_]^4+^ [2]catenane 75, in which two figure-of-eight macrocycles, each formed by joining two ligands together with a Pd^II^ vertex, interlock with each other *via* aromatic stacking between pyridyl and phenylene moieties, while other pyridyls reside in the cavities of the peptidic macrocycles.

## 3. Cages with peptides as auxiliary units

The integration of peptides with rigid aromatic scaffolds is an alternative approach to address the entropic and flexibility challenges of making cages with only peptides as backbones. Rigid aromatic platforms provide geometric preorganisation by constraining spatial arrangements and minimising conformational freedom, facilitating assembly through metal coordination,^69,70^ covalent bond formation,^71,72^ dynamic covalent chemistry,^73,74^ and hydrogen bonding networks.^75,76^ These scaffolds also provide chemically accessible sites for peptide attachment, enabling systematic incorporation of amino acid functionality without compromising structural integrity.

This section examines five primary binding modes for peptide-functionalised cages: metal coordination, covalent assembly, dynamic covalent linkages, hydrogen bonding networks, and hybrid approaches combining multiple interactions. Each strategy offers distinct advantages for incorporating biological functionality while maintaining structural control through rigid aromatic preorganisation.

### 3.1 Metal-coordination cages

**Fig. 6:**
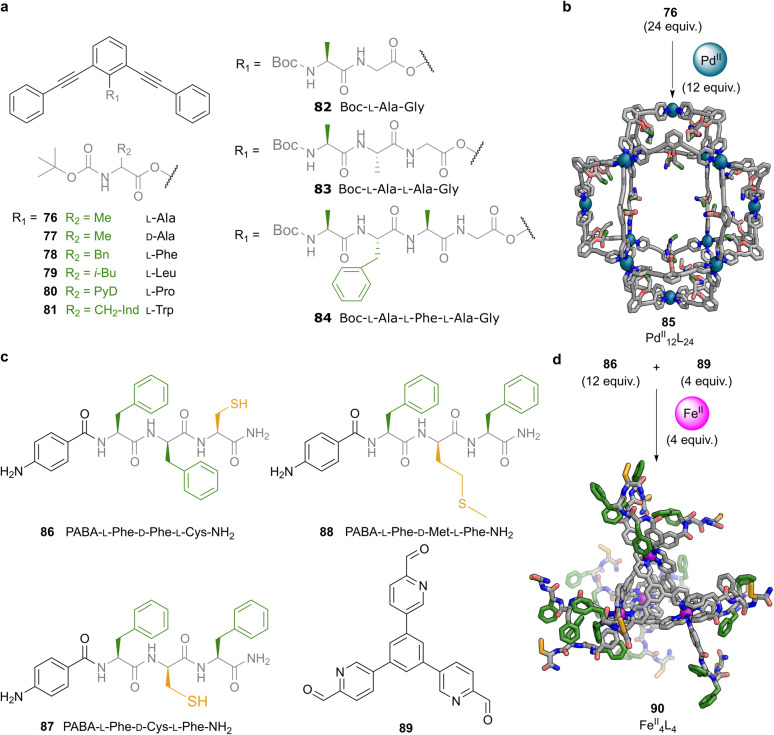
Peptide-decorated metal–organic cages assembled from functionalised aromatic scaffolds. (a) Aromatic bent ligands based on a bis(pyridylethynyl)benzene core were functionalised by esterification at the central arene with aliphatic amino acids and short peptides (76–84). Peptide residues ranged from single amino acids to tetrapeptides and were N-terminally protected to prevent undesired coordination. (b) The assembly of 12 equiv. of Pd^II^ and 24 equiv. of ligand bearing Boc–l-Ala (76) yields a Pd^II^_12_L_24_ cuboctahedral cage (85) displaying 24 amino acid residues on its interior surface. (c) Structures of heterochiral tripeptide ligands 86–88, each incorporating two phenylalanine residues and either cysteine or methionine, alongside the rigid tritopic aromatic aldehyde 89 used in cage formation. Peptides were functionalised with a *p*-aminobenzoyl (PABA) group at the N-terminus and amidated at the C-terminus. (d) The subcomponent self-assembly of 12 equiv. of 86, 4 equiv. of aldehyde 89, and 4 equiv. of Fe(NTf_2_)_2_ in acetonitrile yields Fe^II^_4_L_4_ tetrahedral cage 90. Colour code for rendered structures: Peptide backbone and aromatic scaffold = grey, N = blue, O = red, S = yellow, Pd = teal, Fe = magenta.

In pioneering work nearly two decades ago, Fujita and co-workers exploited the directionality of the coordination bond to synthesise a large [Pd^II^_12_L_24_]^24+^ cuboctahedral metal–organic cage in which peptide moieties were appended to a bent bis(pyridylethynyl)benzene ligand scaffold (Fig. 6a).^69^ The aromatic ligand was functionalised *via* amide coupling at the central aromatic core with different aliphatic amino acids and peptides of up to four residues (76–84). These ligands assembled into cages where all pendant groups were either Boc-protected or acetylated at the N-terminus to prevent undesired coordination to the metal centres. The resulting structures displayed internal surfaces decorated with 24 to 96 amino acid residues, generating confined chiral environments. The [Pd^II^_12_L_24_]^24+^ cage 85 derived from Boc–l-Ala ligand 76 was studied in detail to probe the transfer of stereochemical information from peptide to cage framework (Fig. 6b). CD spectroscopy revealed a Cotton effect for cage 85 approximately 30 times stronger than that of the free ligand at equimolar concentrations.

Notably, cages assembled from amino acid enantiomer 77 exhibited mirror-image Cotton effects, demonstrating that the chirality of the amino acid residues effectively dictated the handedness of the entire cage structure. This chiral signal amplification was attributed to the collective twisting of the ligand framework induced by the peptide stereocentres.^77^ Building on this design, the authors prepared mixed-ligand cages incorporating Boc–l-Phe (78) and Boc–l-Pro (80) residues, to introduce additional functionality into the confined space. The phenylalanine residues provided aromatic surfaces capable of stacking interactions and enhanced hydrophobic contacts, potentially improving guest-binding selectivity. In contrast, the inclusion of proline residues was intended to confer organocatalytic activity. Interestingly, when the longer peptide l-Ala–l-Val–l-Phe–l-Ala–l-Gly was introduced, cage formation was no longer observed, likely due to steric hindrance within the cavity. This result pointed to a volumetric threshold of approximately 100 amino acid residues per cage, a value comparable to the size of small proteins.

Further investigations revealed that cage 85 self-assembles into hollow spherical aggregates of ∼38 nm radius through electrostatic interactions and aromatic stacking.^78^ These “blackberry” structures form when nitrate counterions reduce intercage repulsion, leading to a monolayer morphology reminiscent of viral capsids.^79,80^

Liu and co-workers demonstrated that racemic mixtures of 76- and 77-based cages undergo chiral self-sorting into enantiopure aggregates.^81^ Chiral counterions such as deprotonated Boc–d/l-Ala modulate assembly in an enantioselective manner. Matched chirality pairs show weak interactions, allowing efficient nitrate binding and aggregation. Amino acid identity proved crucial, as Val- and Leu-functionalised cages showed slower aggregation and reduced chiral discrimination with hydrophobic counterions, suggesting a steric/hydrophobic threshold where non-specific interactions dominate over stereoselective contacts.^82^

In summary, this family of cages demonstrates the central role that hydrophobic amino acid residues can play in functional metal–organic cages. Their incorporation initially served to determine the chirality of the metal–ligand framework, and they proved to be essential in modulating higher-order assembly processes too. The ability of these cages to form hierarchical supramolecular structures, particularly those reminiscent of viral capsids, points to fruitful avenues for bioinspired materials development. Furthermore, the observation that subtle changes in amino acid identity can control chiral recognition provides a platform for probing fundamental aspects of homochirality, with implications for both catalysis and molecular evolution.

Ligands based on bent bis(pyridylethynyl)benzene derivatives have enabled the construction of other discrete architectures, such as Pd^II^_2_L_4_ cages, due to predictable coordination geometry and rigidity.^83^ The ligand scaffold is synthetically accessible and modular, as the central aromatic core can be readily functionalised through reactions such as palladium-catalysed cross-coupling or amide coupling. This synthetic flexibility has enabled the introduction of various functional groups, including bioactive peptides, while preserving the coordination geometry required for cage formation.

Notably, several recent studies have employed this ligand framework to prepare metallacages functionalised with peptides that promote biological targeting, such as integrin-binding^84^ and blood–brain barrier-penetrating sequences.^85^ These examples will be discussed in greater detail in the dedicated section on applications below, where the use of peptide-functionalised cages for biomedical purposes is examined more thoroughly.

Minimalistic peptides are also attractive building blocks thanks to their ability to form supramolecular nanostructures and gels.^86^ Their ability to self-assemble under mild conditions has enabled their application in areas ranging from catalysis^87^ to biomedicine.^88,89^ These peptides are not only synthetically accessible and low-cost, but they can also be produced in both solution and solid phases at gram scale, often using green coupling agents.^90,91^ Among minimalistic peptides, tripeptides stand out as an optimal subclass because they retain a compact structure while providing high binding specificity and remaining relatively simple to synthesise. Their short length allows them to achieve affinities close to the ideal binding free energy proposed for biological recognition, striking a balance between selectivity and structural economy. This versatility makes tripeptides appealing not only as recognition elements but also as tuneable building blocks for supramolecular architectures.

Specific design motifs can be readily incorporated into tripeptide sequences to promote self-assembly. A prominent example is the Phe–Phe motif, known for its strong tendency to drive aggregation into a variety of nanostructures.^92–94^ In parallel, chirality plays a key role in guiding the supramolecular organisation. The alternation of l- and d-amino acids within tripeptides promotes an amphiphilic β-strand-like conformation in which hydrophobic sidechains and polar backbone elements are projected on opposite sides of the molecule, enabling hierarchical assembly.^18^

These peptide design principles have recently been applied to the construction of [Fe^II^_4_L_4_]^8+^ tetrahedral cages with heterochiral tripeptides 86–88 appended at the vertices (Fig. 6c).^70^ The three peptides selected for this study were designed to contain two phenylalanine residues and one sulfur-containing amino acid, either methionine or cysteine. The peptides were further functionalised by amidation at the C-terminus, to avoid metal coordination by the carboxylic acid group. They were also functionalised by introducing a PABA group at the N-terminus to facilitate imine bond formation.

The condensation of these tripeptides with rigid tritopic aldehyde ligand 89 around Fe^II^ template ions in acetonitrile at 60 °C for 18 hours yielded well-defined [Fe^II^_4_L_4_]^8+^ cages. ^1^H NMR analysis revealed that the cages exist in solution as a mixture of two diastereomers with opposite handedness at the Fe^II^ vertices, designated as Δ_4_ and Λ_4_ configurations.^95^ One diastereomer formed preferentially, and the diastereomeric excess was attributed to stereochemical induction by the enantiopure peptide ligands, which transmit their handedness to the metal centres during the subcomponent self-assembly process.^96,97^ CD spectroscopy confirmed which diastereomer predominated by revealing Cotton effects in both the near UV (210 to 410 nm) and visible (490 to 630 nm) regions, corresponding to π-to-π* and metal-to-ligand charge transfer (MLCT) transitions, respectively. The sign of the MLCT Cotton band correlated with the dominant metal handedness, with Λ-handed cages observed for the LDL peptides. As expected, inversion of the peptide chirality, DLD, led to opposite Cotton effects.

Interestingly, differences in the near UV region of the CD spectra, associated with the secondary structure of the peptide arms, highlighted conformational differences between the cages. Peptides containing two phenylalanine residues gave rise to spectral features consistent with β-strand conformations.^98^ In contrast, the peptide containing methionine showed a distinct signature suggestive of a PP_II_-like arrangement or a type I β-turn.^99^ These structural variations influenced the cages' ability to form metallogels in the presence of a second metal ion, a phenomenon discussed in Section 4 below.

Cyclic peptides offer an alternative scaffold for metal-coordinated cage assembly. Kubik demonstrated that cyclic tetrapeptides comprising l-proline and 3-amino-5-(pyridin-4-yl)benzoic acid subunits assemble into discrete coordination complexes with Pd^II^. Depending on the metal precursor, either a dimetallic Pd_2_L_2_ macrocycle or a trimetallic Pd_3_L_6_ cage forms.^100^

### 3.2 Covalent cages

**Fig. 7:**
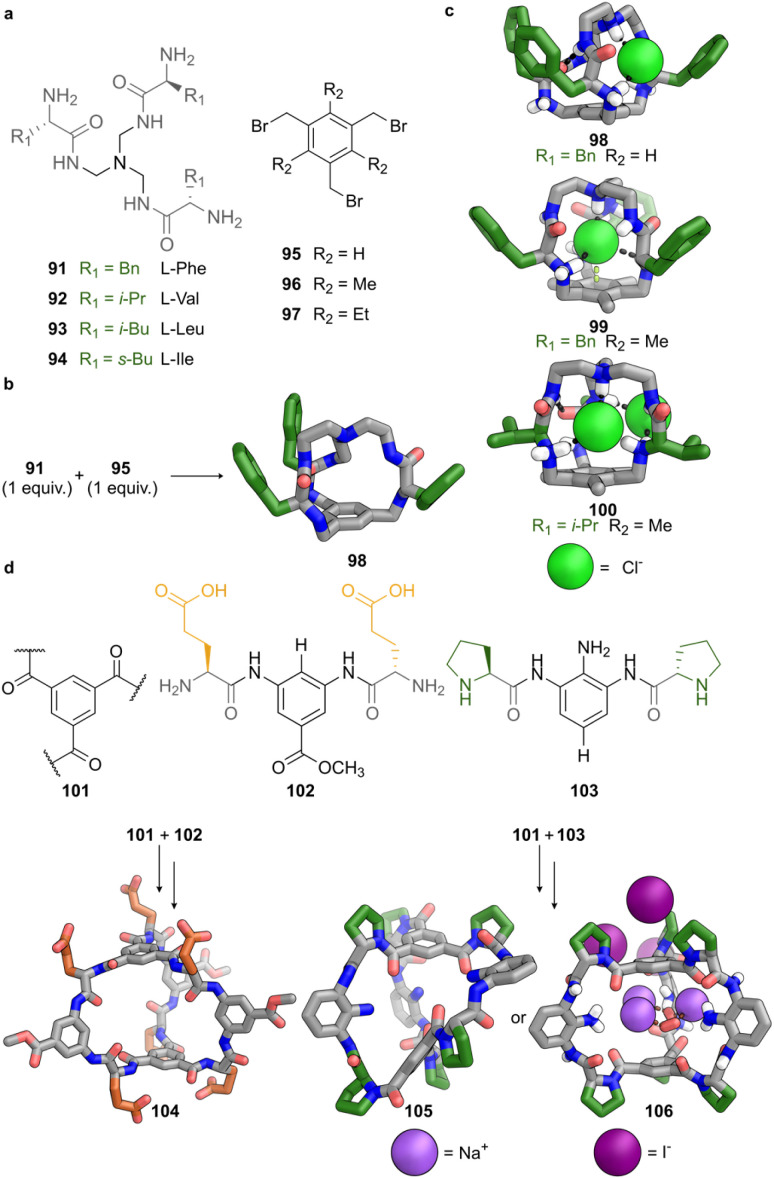
Covalent peptide-based cages formed from tripodal scaffolds and modular aromatic linkers. (a) Structures of tripodal pseudopeptidic building blocks (91–94), derived from triamines functionalised with amino acid residues, and 1,3,5-tris(bromomethyl)benzene scaffolds 95–97 used to promote cage formation *via* S_N_2 substitution. (b) Assembly of cage 99 through the reaction between 1 equiv. of 93 and 1 equiv. of 96. (c) SCXRD structures of cages 98–100, showing distinct chloride binding modes influenced by scaffold geometry and side-chain identity. Cage 98, derived from 95, exhibits a collapsed conformation with externally bound chloride. Cage 99 features a well-defined cavity with internally coordinated chloride. Cage 100, based on Val-derived tripodal ligand 92, displays dual chloride binding with one anion partially encapsulated and one bound externally. (d) Covalent cages 104–106 formed from benzene tricarboxamide caps (101) linked by peptide-based arms bearing Glu (102) or Pro (103) residues through DAB linkers. Cage 104 adopts a *C*_3_-symmetric conformation with a hydrophilic cavity. Cage 105 collapses in solution but undergoes guest-induced expansion upon crystallisation with NaI, yielding cage 106 with a hydrated sodium cluster bound within the re-opened cavity. Colour code: peptide backbone and aromatic scaffold = grey, N = blue, O = red, Cl = lime green, Na = violet purple, I = deep purple, hydrogen bonds = dashed black.

Covalent bonds offer a versatile platform for constructing synthetic cages with high stability and structural definition.^71^ In 2012, Alfonso and co-workers reported a covalent strategy in which tripodal pseudopeptidic building blocks 91–94, obtained from aliphatic triamines functionalised with amino acid residues, react with symmetrical 1,3,5-tris(bromomethyl)benzenes (95–97) *via* three consecutive S_N_2 reactions. The aromatic core acts as a rigid cap that defines the cage structure (Fig. 7a). The reaction, promoted by bromide anions, yields [1 + 1] pseudopeptidic cages (98) when the geometry of the macrocyclic intermediate allows efficient intramolecular closure (Fig. 7b). Molecular dynamics simulations revealed that the pseudopeptidic tripods adopt a preorganised concave conformation, stabilised by hydrogen bonding between amide and ammonium groups and the bromide ion itself. The geometry at the final S_N_2 substitution step governed the formation of either a [1 + 1] pseudopeptidic cage or a [2 + 2] dimer. Selective [1 + 1] cage formation occurs only when the N⋯CH_2_Br distance in the macrocyclic intermediate is sufficiently short to favour intramolecular attack. This distance depends on the nature of the central scaffold. Aliphatic scaffolds, such as tren, imposed the geometric constraints required for efficient cyclisation. Aromatic scaffolds do not preorganise the arms as effectively, resulting in competing intermolecular pathways and inseparable mixtures of [1 + 1] and [2 + 2] cages.

This strategy was extended to incorporate different amino acid residues, revealing how side-chain properties influence structure and binding. A comparison between two Phe-based cages (98 and 99) illustrates the key role of substituents at the tripodal aromatic platform. SCXRD analysis of 98, derived from 95 (R_2_ = H) revealed a collapsed, less symmetric conformation in which chloride is bound externally to the cavity, interacting with ammonium and amide NH groups (Fig. 7c). By contrast, 99, derived from methyl-substituted 96 (R_2_ = Me), displays a tightly encapsulated chloride, coordinated in an approximately tetrahedral arrangement by four ammonium groups and positioned directly above the centroid of the aromatic core, suggesting stabilisation through anion-π interactions (Fig. 7c). The enhanced binding affinity observed for 99 was not attributed to stronger anion-π interactions, but to steric effects reducing solvation of the ammonium groups, thereby improving accessibility for chloride coordination.

Cage 100, containing Val-derived tripodal ligand 92, exhibited a different behaviour, adopting a dual-binding mode. One chloride ion is partially included at the cavity entrance, interacting with two secondary and one tertiary ammonium groups. A second chloride is bound externally *via* hydrogen bonds to amide and ammonium NH groups (Fig. 7c). This dual mode reflects the reduced encapsulation efficiency of aliphatic cages and greater involvement of the hydrogen-bonding network in guest recognition.

Solution-phase studies further confirmed the lower chloride affinity of aromatic cages 98 and 99, in contrast to the ones derived from aliphatic tripodal ligands (92, 93, or 94). The decreased affinity in the Phe-based cages was attributed to steric shielding by aromatic side chains, hindering access to key binding functionalities. The more compact and accessible environments of the aliphatic cages enhance anion recognition by reducing steric interference.

These findings demonstrate how peptide-derived building blocks, when combined with suitably preorganised scaffolds, can yield defined covalent cages that exhibit selective guest binding. The approach offers excellent synthetic control but also highlights inherent limitations. Small variations in scaffold or side-chain geometry can result in unpredictable product distributions or reduced binding performance. A key challenge remains to define general design rules that reliably translate molecular components into desired three-dimensional architectures.

An alternative approach, reported in 2025 by the Cai and Ke groups, introduced covalent tripodal cages constructed from rigid aromatic caps and modular peptide arms.^72^ Each cage consists of two benzene tricarboxamide units, 101, that define the top and bottom planes of the structure. These are connected by three arms each comprising two amino acid residues linked through a central 1,3-diaminobenzene (DAB) unit (Fig. 7d). The angular constraint imposed by the DAB is essential for macrocyclisation, promoting preorganisation and convergent alignment of the arms. This design offers dual modularity. The DAB unit can be combined with a variety of amino acids, from charged residues such as glutamic acid (102) to aliphatic ones such as alanine and proline (103), providing control over backbone rigidity, hydrophilicity and hydrogen bonding. In addition, the DAB scaffold allows peripheral functionalisation. The introduction of bulky dicyclohexyl substituents on the DAB linkers increases steric pressure on the arms, enabling fine-tuning of cavity shape, hydration state and guest accessibility.

Cage 104 adopts a *C*_3_-symmetric structure in solution, as evidenced by sharp singlets in the ^1^H NMR spectrum. SCXRD analysis revealed a well-defined cavity in which the opposing 101 caps are separated by 9.0 and 9.4 Å in the distinct solid-state conformations. The side chains of 102 are engaged in hydrogen bonding with water molecules. The three arms adopt slightly different conformations, reflecting minor variations in carbonyl and amide orientations, consistent with dynamic averaging observed in solution. These observations demonstrate how a flexible, hydrophilic residue supports the formation of a compact, yet adaptive, cavity.

Replacement of glutamic acid with proline leads to pronounced structural changes. Cage 105, incorporating rigid residues of 103, shows desymmetrisation in solution. SCXRD of 105 shows a collapsed conformation, with 101 caps arranged orthogonally and separated by only 4.8 Å. The cavity is occluded by a hydrogen-bonded network of water molecules and stabilised by intramolecular hydrogen bonds involving terminal amines and backbone NH groups. Crystallisation in the presence of NaI induces a guest-dependent rearrangement. The SCXRD structure 106 displays a symmetric conformation, with 101 caps aligned in parallel and separated by 6.7 Å, encapsulating a hydrated sodium cluster coordinated by carbonyl and water ligands. This behaviour demonstrates the ability of 105 to undergo guest-induced conformational transitions between collapsed and expanded states.

This modular approach demonstrates how peptide sequence, linker geometry, and peripheral substitution can be strategically combined to fine-tune the properties of covalent cages. The capacity to modulate cavity shape, rigidity and hydrophilicity through targeted chemical modifications represents a strength of this design. At the same time, these systems exemplify the inherent complexity of molecular engineering: even small variations in side-chain polarity or hydrogen-bonding potential can induce significant changes in structure and function. The sensitivity of cage assembly to these parameters underscores the importance of integrating detailed experimental characterisation with rational design principles.

Overall, the systems developed by Alfonso and by Cai and Ke illustrate two distinct but complementary strategies for constructing covalent peptide-based cages. The former exploits intramolecular S_N_2 reactions and scaffold-guided preorganisation to promote efficient macrocyclisation. The latter employs rigid aromatic platforms and bent linkers to generate structurally diverse and tuneable architectures. Both approaches highlight the potential of biologically inspired building blocks for selective molecular recognition, but also expose the challenges of achieving precise conformational control and cavity definition in aqueous environments. Addressing these challenges will require a deeper understanding of how scaffold geometry, side-chain interactions and solvation collectively govern the assembly and behaviour of peptide-derived cages.

### 3.3 Dynamic-covalent cages

Although irreversible covalent bonds afford exceptionally stable cages, their assembly typically requires lengthy multistep syntheses, low overall yields, and the use of highly preorganised ligands to suppress oligomer formation. To overcome these limitations, dynamic covalent chemistry (DCC) provides an alternative strategy, exploiting reversible bond formation under thermodynamic control to enable self-correction and favour the most stable product.

**Fig. 8:**
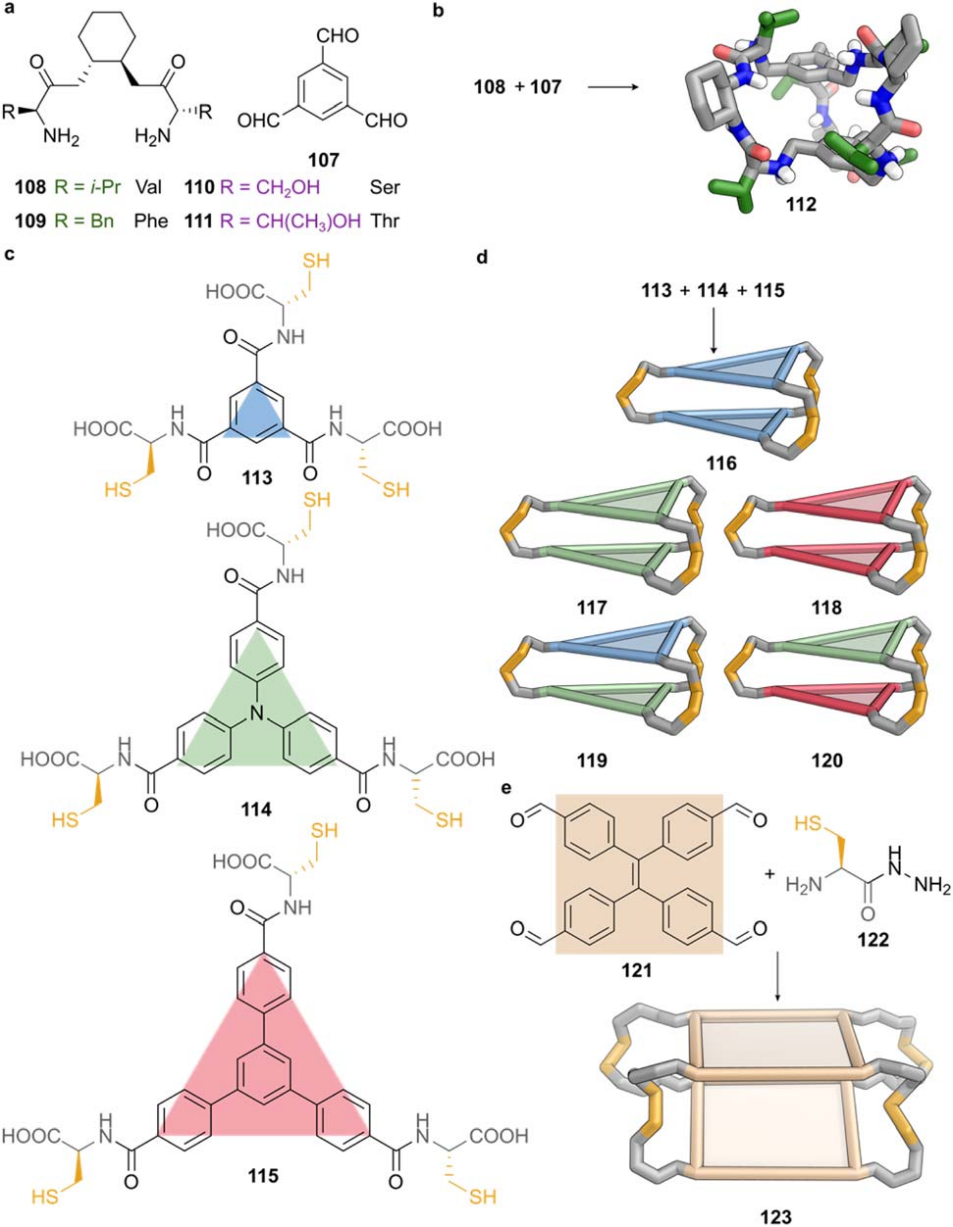
Peptide-derived cages constructed through dynamic covalent strategies. (a) Dynamic-covalent cage assembly from benzene-1,3,5-tricarbaldehyde (107) and a series of *C*_2_-symmetric bis(amidoamine) linkers (108–111), each incorporating a *trans*-1,2-cyclohexane scaffold and a distinct amino acid residue: valine (108), phenylalanine (109), serine (110), or threonine (111). (b) Self-assembly of 1 equiv. of 107 and 1.5 equiv. of 108 under reversible imine formation followed by *in situ* reduction yields cage 112. SCXRD of 112 reveals chair conformations of the cyclohexane ring, with outward-projecting isopropyl groups. (c) Aromatic tritopic building blocks of different sizes (113–115) and bearing Cys moieties leads to *D*_3_-symmetric homoleptic cages *via* direct oxidation in water at pH 8. (d) Dynamic disulphide exchange strategy using 113 with 114 or 114 with 115 yields mixtures containing both homodimeric (116–118) and heterodimeric cages (119–120). By contrast, the pair 113 and 115 fails to form heterodimers due to unfavourable geometry. Structures 116–120 are shown as cartoons, where coloured triangles represent the shapes and relative sizes of the aromatic platforms. (e) Assembly of cage 123*via* dual dynamic covalent reactions, acylhydrazone linkages between TPE-based aldehyde 121 and cysteine-hydrazide 122, along with disulphide bonds formation, yield a closed tetrapodal cage. The resulting restriction of TPE rotation induces aggregation-induced emission. Cage 123 is represented as a schematic square reflecting the geometry of the tetratopic core.

Simultaneously with their work on tripodal cages described above, Alfonso and co-workers developed a complementary strategy based on DCC to assemble pseudopeptidic cages through reversible imine formation, followed by reduction to amine linkages.^73^ In this approach, benzene-1,3,5-tricarbaldehyde (107) generates both the top and bottom platforms of the cage, while the three aldehyde groups are connected by *C*_2_-symmetric bis(amidoamine) linkers (Fig. 8a). Each linker incorporates an amino acid residue and either a rigid *trans*-1,2-cyclohexane or a flexible ethylene spacer. The conformational constraints imposed by the cyclohexane moities promote preorganisation, allowing the assembling system to self-correct through reversible imine exchange under thermodynamic control, and afford a single *D*_3_-symmetric hexa-imine intermediate. Subsequent *in situ* reduction using borane–pyridine yields the corresponding pseudopeptidic amine cage in overall yields ranging from 30 to 60%, depending on the steric and electronic properties of the incorporated amino acid (Fig. 8b).

SCXRD structure of cage 112, containing Val residues from subcomponent 108, reveals that all three cyclohexane rings adopt chair conformations, with each amide NH positioned *anti* to the adjacent C–H (Fig. 8b).^101^ The isopropyl side chains adopt pseudo-equatorial orientations and project outward from the cage surface, while the cavity accommodates four perchlorate anions. These anions form hydrogen bonds with both the ammonium groups and the amide NH donors, reflecting an inherent affinity of this architecture for anions, and underscoring how side-chain disposition influences cavity accessibility and binding capacity.

In contrast, cages derived from flexible ethylene-linked bis(amidoamines) require benzene-1,3,5-tricarboxylate as a template to form the same *D*_3_-symmetric architecture efficiently.^73^ Modelling suggests that the template engages in convergent hydrogen bonding with amide donors in the cavity, aligning the arms and facilitating cage closure. In the absence of the template, greater conformational freedom reduces the yield and selectivity of cage formation, highlighting the critical role of linker geometry and preorganisation in dynamic covalent assembly.

This methodology accommodates a diverse range of amino acids, including aliphatic, aromatic, and polar residues, enabling fine tuning of the steric environment, hydrogen-bonding landscape, and hydrophilicity of the cages.^101,102^ Variations in side-chain functionality allow modulation of complementarity and stereoselectivity, with serine (110) and threonine (111) introducing additional hydrogen-bond donors that enhance interactions with polar guests, such as dipeptides. Aromatic residues expose their π-surface area within the cavity, supporting favourable stacking interactions with aromatic portions of guest molecules. This modularity establishes these pseudopeptidic cages as an expandable library, in which backbone geometry and side-chain diversity can be systematically exploited to tailor recognition properties.

This modular, self-correcting, and template-sensitive imine-based approach allows systematic variation of cavity properties, but binding affinities remain moderate in mixed organic solvents, which could limit performance in fully aqueous or competitive environments. The synthetic protocol requires chromatographic purification and counterion exchange, adding procedural complexity. The rigid *D*_3_ symmetry further constrains the accessible guest space, favouring small anionic or peptidic targets. Nevertheless, these pseudopeptidic cages demonstrate how amino acid functionalities and aromatic frameworks can be combined to tune cavity polarity, rigidity, and selectivity.

Disulfide exchange is another established dynamic covalent strategy for synthesising a wide variety of supramolecular architectures. A key study published in 2012 by Sanders and Stefankiewicz demonstrated the creation of a dynamic combinatorial library (DCL) of cages based on disulfide bonds.^74^ The system used a tritopic building block derived from 1,3,5-trisubstituted benzene bearing three cysteine units (113) (Fig. 8c), combined with the ditopic bridging linker 3,5-dimercaptobenzoic acid. The system in water at pH 8 without any template generated a mixture of cyclic trimers, tetramers, and a dimeric cage. Upon addition of protonated polyamines, the library distribution was amplified towards specific multicomponent cages depending on the nature of the templating polyamine. Linear polyamines bearing multiple ammonium groups, such as spermine and triethylenetetramine, most effectively promoted the formation of larger, defined cages. The distribution of amplified species was determined by the length, flexibility and charge density of the polyamines.

While this approach enabled the generation of structurally complex architectures, it suffered from intrinsic limitations. The DCL could not be amplified towards a single dominant species, and the resulting mixtures were challenging to purify.

In 2021, Stefankiewicz expanded the disulfide cage strategy by incorporating larger aromatic platforms into the same framework (Fig. 8c).^103^ The original tritopic building block (113) was retained, but additional aromatic units were introduced into compounds 114 and 115, to incrementally increase the size of the rigid platform. The cysteine residues served a dual role, providing both disulfide-forming thiols, and terminal carboxylate groups that ensured water solubility. In this system, no ditopic linker was employed, and direct oxidation at pH 8 in water yielded exclusively homoleptic cages with *D*_3_ symmetry. While no X-ray crystal structures were obtained, ^1^H NMR spectra showed upfield shifts for aromatic resonances, consistent with close spatial proximity of the aromatic platforms, and deshielding of the cysteine α and β protons indicated their exposure at the periphery of the cages. The study also explored heterodimeric cage formation by mixing pairs of building blocks. Combinations of 113 with 114, and of 114 with 115, yielded three products each: the homodimers, 116–118, and the corresponding heterodimers, 119 and 120 (Fig. 8d). By contrast, the combination of platforms 113 and 115 produced only the homodimeric species without detectable heterodimer (Fig. 8d). This selectivity was rationalised in terms of two geometric parameters, the rotation (*θ*) and trapezoidal (*ζ*) angles, determined by molecular modelling. *θ* quantifies the degree of twist required to align two platforms for disulfide bond formation, with values above 80° preventing cage closure, as in the case of the heterodimer composed of 113 and 115 (*θ* > 100°). *ζ* defines the angle between the plane of the aromatic platform and the cysteine arm, reflecting the distortion required to bridge the platforms. In this case, values exceeding 120° prevent cage formation, as with the heterodimer from 113 and 115, characterised by a size mismatch between platforms.

These examples confirm that disulfide-based chemistry provides a modular approach for constructing larger peptide-derived assemblies. Nevertheless, directing the dynamic combinatorial library towards a single thermodynamically favoured product remains a challenge. For this reason, strategies combining disulfide exchange with orthogonal dynamic processes, such as acylhydrazone formation, have emerged as promising routes to improve fidelity and control over the outcome of self-assembly.

In 2017 Ulrich and Stefankiewicz described a tetrapodal cage constructed from 1,1,2,2-tetraphenylethene (TPE) tetracarboxaldehyde core 121 and cysteine-hydrazide linker 122 (Fig. 8e).^104^ The four benzaldehyde groups and phenyl rings of 121 can rotate freely in solution, rendering the precursor non-emissive. Upon cage formation, these rotations become restricted, resulting in aggregation-induced emission that enables real-time monitoring of the assembly process by fluorescence. In this design, each hydrazide condenses with an aldehyde to form eight acylhydrazone linkages, stabilised by internal hydrogen bonding that zips the structure into a closed and rigid shell. Simultaneously, the oxidation of cysteine thiols formed two disulfide bonds, which preorganise the four TPE units into a stacked, face-to-face arrangement. This combination of stacking and hydrophobic effects directs the dynamic library towards the exclusive formation of a single, fully closed tetrapodal cage 123 (Fig. 8e).

This method offers functionalisation opportunities, as the amino termini of the cysteine residues remain accessible for post-synthetic modification.^104^ This was demonstrated by coupling hydrophilic units, such as 2-(2-methoxyethoxy)acetic acid, and hydrophobic groups, such as 2-ethylhexanoic acid, which modulate cage solubility in aqueous media or organic solvents such as chloroform. DMSO was required in all cases to facilitate thiol oxidation and ensure cage formation. This solubility-tuning expands the operational scope of these doubly dynamic covalent architectures, while retaining their characteristic emission properties, with potential applications as responsive fluorescent materials.

### 3.4 Hydrogen-bond directed cages

**Fig. 9:**
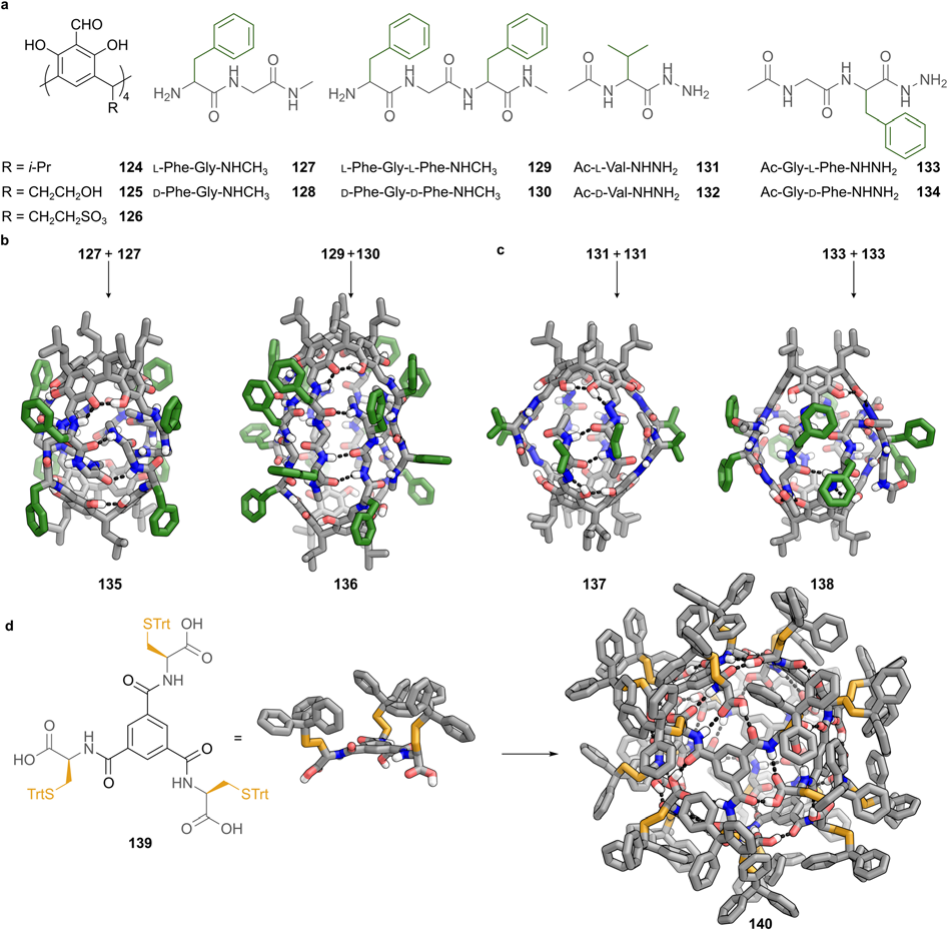
Peptide-derived cages assembled *via* dynamic covalent bonds and hydrogen bonding. (a) Tetraformylresorcin[4]arene scaffold 124 and peptide-based ligands employed for cage formation. Imine-linked systems utilise dipeptides 127–128 and tripeptides 129–130 with terminal amines, while hydrazone-linked systems employ hydrazide derivatives of valine 131–132 and Phe–Gly 133–134. (b) Imine-based cages assembled through condensation of amine-functionalised peptides 127–130 with scaffold 124. Cage 135, obtained from homochiral dipeptides, adopts a symmetric dimeric architecture sealed by twelve hydrogen bonds. Cage 136 forms preferentially from racemic tripeptides, showing social self-sorting into heterochiral dimers stabilised by 24 hydrogen bonds arranged in an antiparallel β-sheet-like pattern. (c) Acylhydrazone-based cages generated by condensation of hydrazide-functionalised ligands 131–134 with scaffold 124. Homochiral ligands yield discrete, rigid cage 137, derived from valine ligand 131, which adopts a partially open conformation with outward-facing isopropyl groups and a solvent-accessible cavity; 138, assembled from Phe–Gly ligand 133, displays a more compact, closed conformation with the peptide arms folding tightly to exclude solvent. Both cages are stabilised by a continuous seam of 16 antiparallel hydrogen bonds, and only homochiral assembly is observed due to the planar geometry of the hydrazone linkage. (d) Structural features of monomer 139 employed in the formation of hydrogen-bonded octameric cage 140. Molecule 139 consists of a central 1,3,5-trisubstituted benzene ring bearing three cysteine residues. Each cysteine sulphur is protected with a trityl group. SCXRD shows that all trityl groups are oriented on the same face of the aromatic core, enforcing a directional arrangement in which the three carboxylate groups project outward from the opposite face. This spatial preorganisation facilitates cooperative hydrogen bonding and promotes cage assembly. SCXRD of cage 140 assembled exclusively through hydrogen bonding. Eight units of 139 form a spheroidal architecture featuring 48 cooperative hydrogen bonds. The resulting structure displays *D*_2_ symmetry with partially open pores at the equator and poles, enclosing a central cavity of ∼1700 Å^3^. Colour code: peptide backbone and aromatic scaffold = grey, N = blue, O = red, S = yellow, hydrogen bonds = black.

Hydrogen bonding offers an alternative strategy for controlling peptide-based cage architectures by guiding strand alignment and registry within confined geometries. The pseudopeptidic cages developed by Szumna and co-workers are constructed on tetraformylresorcin[4]arene scaffolds (124) that present four formyl groups arranged symmetrically around a concave aromatic platform (Fig. 9a).^75,105^ These scaffolds provide a rigid aromatic foundation capable of engaging multiple peptide-derived ligands to form discrete cages in solution and in the solid state. Two distinct types of dynamic covalent bonds have been employed to assemble these structures. Imine bonds are formed *via* condensation with amines, whereas acylhydrazone bonds are formed *via* condensation with hydrazides (Fig. 9a). The difference between these linkages has profound consequences for configurational flexibility, geometric adaptability and self-sorting behaviour.

Imine bonds formed from resorcinols allow tautomerism between enol–imine and keto–enamine forms. The aldehyde groups of the resorcinarene scaffolds predominantly adopt an enolic configuration, while the imine linkage itself can tautomerise locally. The keto–enamine tautomer locks the C=N bond into a planar, rigid geometry coplanar with the aromatic ring, whereas the enol–imine tautomer may adopt a non-coplanar geometry. This tautomeric flexibility allows structural adaptation during self-assembly that can relieve steric strain and optimise stacking or CH–π interactions, contributing to the configurational plasticity that enables sequence-dependent sorting behaviour.

The amino acid sequences selected for these studies were carefully designed to test how peptide length, chirality, and side-chain functionality influence self-assembly. In the imine-linked series, alternating hydrophobic and hydrophilic residues such as phenylalanine and glycine were used to mimic β-sheet-like hydrogen bonding motifs. These residues generate amphiphilic strands capable of forming antiparallel arrangements. For dipeptides, such as l-Phe–Gly–NHCH_3_127 or its enantiomer 128, sequence parity ensures alignment of hydrogen-bond donors and acceptors without inversion of strand orientation. Crystallographic analysis of homochiral cage 135 revealed a symmetric architecture sealed by 12 hydrogen bonds (Fig. 9b). The structure displayed asymmetry at the molecular level, where one hemisphere contained rigid keto–enamine linkages coplanar with the resorcinarene core, while the opposite hemisphere exhibited flexible enol-imine linkages with distorted geometries.^75^ This asymmetry reflects the dynamic adaptability conferred by imine tautomerism, allowing the cage to minimise strain, while maintaining a continuous hydrogen-bond network.

When tripeptides, such as l-Phe–Gly–l-Phe–NHCH_3_ (129), were employed, a different behaviour was observed. The odd number of residues necessitates an inversion of strand orientation to preserve antiparallel hydrogen bonding between hemispheres. This inversion can only occur through heterochiral pairing. Solution-phase NMR indicated that racemic mixtures of tripeptides 129 and 130 preferentially formed heterochiral cage 136, with social chiral self-sorting behaviour, as confirmed by SCXRD analysis. Analysis of 136 showed the alternating strands of opposite handedness arranged in an antiparallel fashion, and were stabilised by 24 hydrogen bonds (Fig. 9b).^75^ These results establish a clear sequence-parity-dependent sorting logic, in which homochiral dimers predominate for even-numbered sequences, whereas heterochiral dimers predominate for odd-numbered sequences, driven by the geometric requirements of maintaining a continuous antiparallel hydrogen-bond seam.

Acylhydrazone-linked cages exhibit behaviour distinct from their imine-linked counterparts, owing to the geometric rigidity imposed by the acylhydrazone bond. This bond adopts a *trans* configuration around the N–N linkage and suppresses tautomerism, resulting in a planar, locked geometry that fixes the orientation of the appended peptide strands, constraining the overall architecture. This rigidity eliminates the conformational adaptability seen in imine-based cages, enforcing strict requirements for stereochemistry and strand alignment during self-assembly.

To investigate the implications of this rigidity, Szumna and co-workers systematically examined peptide-derived hydrazide ligands both as pure enantiomers and as racemic mixtures. The first system employed the L-(131) and D-(132) enantiomers of a valine-derived hydrazide ligand (Fig. 9a). Solution-phase experiments demonstrated that racemic mixtures yielded exclusively homochiral cages, indicating that only assemblies comprising identical enantiomers could satisfy the geometric demands of this rigid framework (Fig. 9c). The crystal structure of homochiral cage 137 (Fig. 9c) based on 131 provided structural insight into this behaviour. Structure 137 revealed a symmetric architecture in which two cavitands are joined to form a dimeric cage, sealed by a continuous seam of 16 hydrogen bonds arranged antiparallel around the cage equator.^105^ The valine side chains projected uniformly outward from the cage surface, consistent with minimising steric hindrance and solvent exposure. Notably, the internal cavity of this cage was partially occupied by solvent molecules, reflecting a conformation where the peptide arms did not fully fold inward to exclude the solvent completely.

A second ligand derived from the dipeptide Phe–Gly–NHNH_2_ was studied to assess the influence of peptide length and composition (Fig. 9a). When enantiomerically pure l-Phe–Gly–NHNH_2_ (133) and d-Phe–Gly–NHNH_2_ (134) were employed, narcissistic self-sorting occurred. SCXRD analysis of homochiral cage 138 (Fig. 9c) derived by 133 showed that the cage was sealed by a continuous seam of antiparallel hydrogen bonds. However, in contrast to the valine cage, cage 138 adopted a more tightly folded structure, fully closing the cavity and preventing solvent occupancy. The crystal structure showed the peptide backbones arranged precisely to align donors and acceptors, while the phenyl side chains projected outward from the cage surface. This observation highlights how increasing peptide length and introducing aromatic side chains promotes greater folding and compactness of the cage, even though the rigid acylhydrazone geometry still dictates strict homochiral sorting. Subsequent work further confirmed that similar behaviour can be observed for ligands with longer peptide sequences, tripeptides and tetrapeptides.^106^

These examples demonstrate how dynamic covalent bond types dictate structural flexibility, sorting logic and responsiveness to environmental conditions. Imine-based cages exhibit configurational adaptability, permitting sequence-parity-dependent sorting, whereby homochiral cages are favoured for even-numbered sequences and heterochiral cages for odd-numbered sequences. Acylhydrazone-based cages, by contrast, exhibit rigid geometries that enforce homochiral assembly irrespective of sequence length and require templation for formation in polar solvents. Both architectures exploit β-sheet-like hydrogen-bond motifs but differ in their tolerance of sequence variation, geometric flexibility, and environmental polarity, illustrating the critical role of bond rigidity and peptide design in determining cage structure and function.

One of the major challenges of these hydrogen-bonded cages is their stability in competitive, polar solvents. Assembly in this context can be promoted by using a hydrophobic template that stabilises the supramolecular architecture. Szumna and co-workers expanded their library of cages by using peptides containing polar amino acids, such as glutamate and histidine, and tetraformylresorcin[4]arene scaffolds functionalised with polar (125) or charged residues (126) to promote hydrogen bonding and water solubility. The introduction of hydrophobic guests, such as C_60_, induces the formation of well-defined capsular dimers even in the competitive media of water and DMSO.^107^ The X-ray crystal structure obtained for a valine-derived cage provides a structural model for this family of assemblies, revealing a cavity enclosed by peptide backbones arranged in an antiparallel β-barrel-like configuration around the encapsulated fullerene. In 2017, Stefankiewicz and Sanders reported an octameric cage assembled exclusively through hydrogen bonding, without the need for any template.^76^ The cage comprises eight identical enantiopure 139 building blocks, each consisting of a 1,3,5-trisubstituted benzene core bearing three cysteine residues with thiol groups protected as trityl derivatives. The presence of the bulky trityl groups improves solubility in non-polar solvents and promotes cage stability by creating a hydrophobic outer shell.

X-ray crystallographic analysis revealed that all trityl groups were oriented on the same side of the benzene core, directing the three carboxylate groups in the opposite direction (Fig. 9d). The resulting cage 140 exhibits a spheroidal architecture with approximate *D*_2_ symmetry, enclosing a large central cavity of 1719 Å^3^. The external surface is partially open, featuring four small pores at the equator (∼7.3 × 7.4 Å) and two larger pores at the poles (∼7.4 × 8.7 Å), allowing selective guest access while excluding larger species. The cage is stabilised by 48 cooperative hydrogen bonds formed between the carboxylic acid groups and amide NH groups of adjacent building blocks (Fig. 9d). The internal hydrogen bond distances vary subtly depending on their position. Two aromatic CH protons form relatively short hydrogen bonds (∼2.3 Å), resulting in stronger shielding in the ^1^H NMR spectrum, while a third experiences a longer hydrogen bond (∼2.4 Å) and correspondingly weaker shielding. Similar asymmetry is observed for the amide NH protons, reflecting the geometric constraints imposed by the overall architecture. Solution-phase NMR confirmed that this structure is retained in apolar solvents such as tetrachloroethane, and diffusion-ordered NMR spectroscopy (DOSY) provided a hydrodynamic diameter of ∼18.2 Å, consistent with crystallographic dimensions. The cage demonstrates thermal and chemical robustness, remaining intact from −10 to 105 °C and requiring more than 40 equivalents of triethylamine to disrupt the hydrogen-bond network. This stability arises from the highly cooperative nature of the hydrogen-bonding motif and the geometrical constraints of the architecture.

Granja and colleagues reported another system of hydrogen bonded capsules formed from two α,γ-cyclic octapeptides bearing zinc porphyrin caps attached *via* dynamic hydrazone linkages. The cyclic peptide, containing alternating d-leucine and (1*R*,3*S*)-3-aminocyclopentanecarboxylic acid residues, self-assembles into dimers through hydrogen bonding to form the capsule framework. The resulting *C*_2_-symmetric structure recognises bipyridine guests through coordination to both zinc centres, with optimal binding observed for ligands matching the 15.8–16.6 Å interaction distance.^108^

These findings exemplify how purely hydrogen-bonded assemblies can achieve stability and selective guest encapsulation. The system highlights the potential of cooperative hydrogen bonding as a design principle for robust, preorganised nanostructures, and its architecture offers ample opportunities for further derivatisation or tuning, by varying the peripheral cysteine residues or protecting groups.

## 4. Applications of peptide cages

The incorporation of peptides into cage-like supramolecular structures enables a range of functional properties that have motivated exploration across diverse application areas. Peptides provide an extensive molecular toolkit to introduce and fine-tune chemical functionality and physicochemical properties within otherwise rigid architectures. Their compatibility with aqueous and biologically relevant environments has prompted investigation in biomedical contexts, including targeted drug delivery, gene therapy, biosensing, diagnostic imaging, and therapeutic applications, although most studies to date remain at a proof-of-concept level.

Peptides contribute molecular recognition elements through hydrogen bonding, aromatic interactions, electrostatic forces, and hydrophobic effects, which can be exploited for selective guest binding, sensing, and environmental monitoring. Amino acid side chains enable modulation of host-guest interactions and environmental responsiveness, with ionisable residues allowing pH-dependent behaviour in switchable systems relevant to pathophysiological conditions. The incorporation of chiral residues generates locally asymmetric environments that may influence molecular recognition, while peptide sequence and composition affect structural rigidity or adaptability, with consequences for guest loading, release behaviour, and interactions with biological interfaces. Together, these properties position peptide-incorporating cages as versatile platforms for emerging applications in medicine, molecular diagnostics, environmental sensing, and biotechnology while highlighting the need for further development to translate structural design into robust functional performance.

### 4.1 Drug delivery and ion-transport therapy

**Fig. 10:**
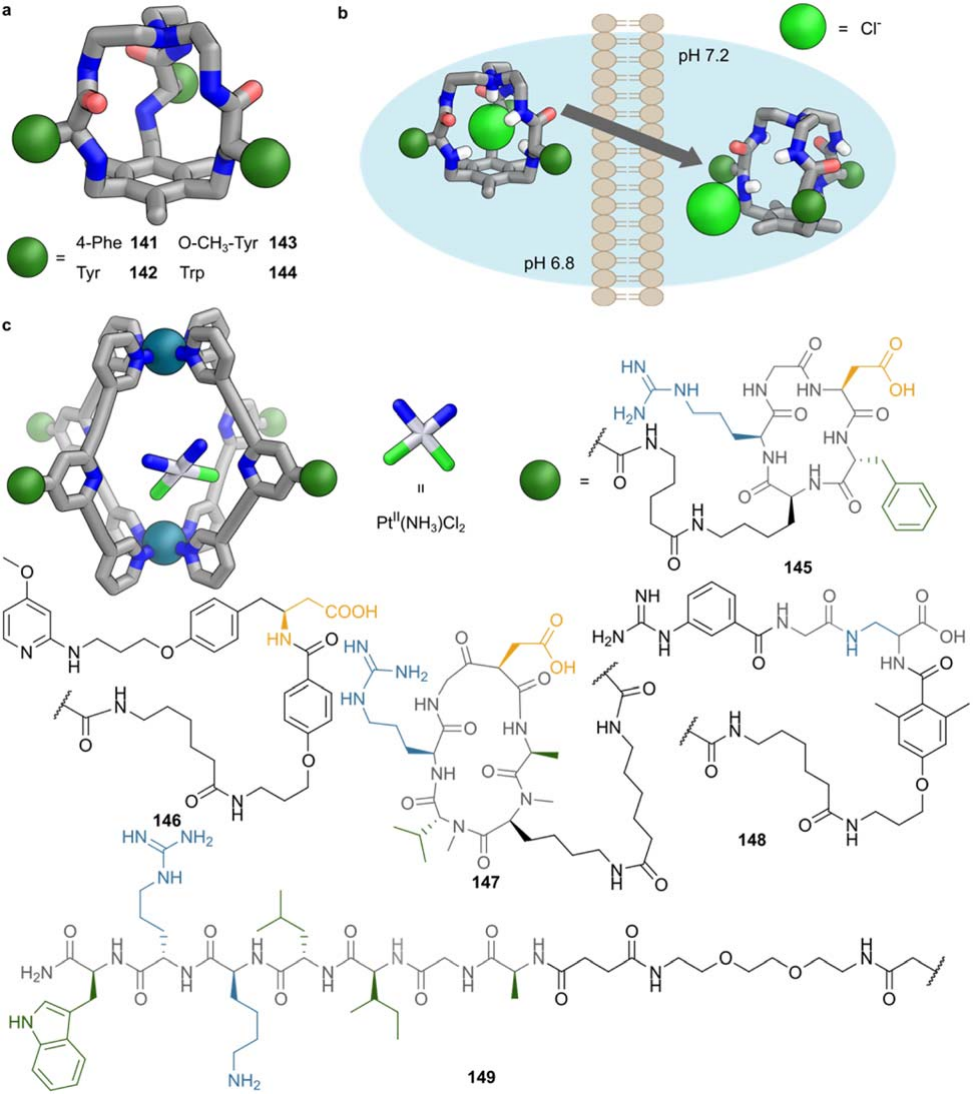
Peptide-functionalised cages for chloride transport, pH-responsive cytotoxicity, and targeted delivery. (a) Representative structure of the tripodal pseudopeptidic cage scaffold common to compounds 141–144. The green spheres represent the aromatic amino acid incorporated into each cage, which varies between 4-F-Phe (141), Tyr (142), *O*-CH_3_-Tyr (143), and Trp (144). These side-chain variations modulate the overall hydrophobicity and membrane interaction profile of the cages, thereby affecting their H^+^/Cl^−^ co-transport efficiency and pH-dependent cytotoxicity. (b) Schematic depiction of pH-triggered H^+^/Cl^−^ symport across phospholipid bilayers. The reverse pH gradient characteristic of tumour microenvironments enables selective chloride release inside cancer cells, inducing cytotoxicity *via* disruption of ion homeostasis. (c) Representative structure of Pd^II^_2_L_4_ cage 145 incorporating different peptide ligands (146–149), denoted as green spheres. Ligands 146–149 display integrin-targeting peptides such as cyclic RGD or α_5_β_1_-binding motifs, enabling selective delivery of cisplatin into tumour cells. Ligand 150 incorporates the PepH_3_ brain-penetrating peptide and enables the corresponding cage to traverse the blood–brain barrier, achieving efficient delivery of diagnostic or therapeutic payloads into neural tissue.

Pseudopeptidic tripodal cage 100, once protonated, was shown to bind up to two chloride anions, enabling anion encapsulation under acidic conditions. This capacity to encapsulate chloride with high affinity under acidic conditions is appealing for developing drug candidates that operate selectively within the acidic microenvironments that are characteristic of solid tumours. This behaviour arises from protonatable binding sites within the cage framework. This property could open the way to ion transport across cell membranes, disrupting cellular homeostasis, affecting membrane potential, and modulating intracellular pH, ultimately leading to cytotoxic responses.^109^ The authors expanded their cage library by exploring aromatic amino acid variants, *i.e.*, 4-F-phenylalanine (141), tyrosine (142), *O*-Me-tyrosine (143), and tryptophan (144) (Fig. 10a).^110^ In these systems, variation of the amino acid side chains modulates hydrophobicity and membrane affinity while preserving the underlying cage architecture.

The amino acid side chains critically influence transport efficiency through an H^+^/Cl^−^ symport mechanism. Compound 141 exhibited superior chloride efflux rates (0.198% Cl per s at pH 6.2) compared to unmodified 100 or 143, correlating with increased hydrophobicity. NMR studies confirmed full lipid phase incorporation while maintaining chloride-binding capacity. Acidic conditions accelerated chloride exchange rates from 0.2 s^−1^ at pH 7.4 to 2.7 s^−1^ at pH 6.2–6.5, enabling selective activation in acidic tumour microenvironments.

This pH-responsive chloride transport capability offers promising anticancer therapeutic applications, although current evidence remains limited to cellular and *in vitro* models, through selective cytotoxicity in tumour microenvironments. Solid tumours exhibit a reverse pH gradient compared to normal tissues, with neutral intracellular pH and a slightly acidic extracellular environment.^111^ This enables the chloride-encapsulated cage to pass through the membrane and selectively release ions inside tumor cells (Fig. 10b). In human lung adenocarcinoma (A549) cells, all cages exhibited enhanced cytotoxicity as extracellular pH decreased from physiological levels. Compound 141 showed a particularly striking pH-dependent activity, with IC_50_ values decreasing from 166 ± 35 µM at pH 7.5 to 29 ± 4 µM at pH 6.2, a five-fold enhancement in cytotoxic potency. Additionally, 141 showed moderate cytocompatibility towards healthy cells at concentrations up to 150 µM. This selectivity reflects the influence of peptide side-chain chemistry on activity under pathological conditions.

Beyond ion transport, bioconjugation of peptides to metal–organic cages has emerged as a strategy for addressing challenges in cancer chemotherapy, particularly integrin receptor overexpression and cisplatin resistance mechanisms. In this context, peptides function as targeting and recognition elements rather than as structural components of the cage framework. Integrins α_v_β_3_ and α_5_β_1_ are overexpressed in various malignant tumours, facilitating tumour angiogenesis, invasion, and metastasis.^112^ This overexpression represents a therapeutic vulnerability exploitable for selective drug delivery. Simultaneously, a major clinical challenge is cisplatin resistance, which is mediated through enhanced DNA repair mechanisms, altered cellular uptake, and increased efflux pump activity.^113^

Encapsulation of cisplatin within metal–organic cages decorated with targeting-motifs offers a potential solution by protecting the drug from deactivation while delivering it selectively to integrin-rich tumour environments. [Pd^II^_2_L_4_]^8+^ cage 145 (Fig. 10c) can be bioconjugated with different peptide-based integrin-binding moieties (145–148), maintaining host-guest chemistry while acquiring selectivity for specific cellular targets.^84^ Here, the rigid aromatic scaffold defines cage geometry, while peptide conjugation introduces biological specificity. Multiple ligand units displayed on the cage scaffold resulted in higher binding affinity than individual free ligands, with 146 demonstrating over a 3-fold improvement in α_v_β_3_ selectivity relative to the reference compound cilengitide.

Peptide conjugation did not compromise structural integrity, as confirmed by ^1^H-NMR spectroscopy showing characteristic downfield shifts of α-pyridinyl protons upon metal coordination. The peptide component ensures binding selectivity through high-affinity recognition of overexpressed integrin receptors. It also contributes to shielding the cage surface, protecting encapsulated drugs from premature release during circulation, and enables controlled release mechanisms that respond to target tissue microenvironments.

Cage 146 (Fig. 10c), when loaded with cisplatin, exhibited improvements in therapeutic selectivity. Cytotoxicity against α_v_β_3_-overexpressing A375 melanoma cells was enhanced 2.1-fold, while showing no increased toxicity against α_v_β_3_-negative A549 cells. Mass spectrometry demonstrated significantly reduced platinum accumulation in healthy liver and kidney tissues for encapsulated cisplatin compared to free drug (*p* ≤ 0.01). These findings suggest that the coordination complex remained intact during biological transit, with the peptide component providing selective targeting while the cage architecture shielded the platinum payload from non-specific interactions. These results indicate that peptide-mediated targeting modulates biodistribution while the cage architecture preserves structural integrity and encapsulation.

The versatility of Pd^II^_2_L_4_ cages was further demonstrated through the functionalisation of a ditopic ligand with a brain-penetrating peptide, PepH3 (149), to traverse the blood–brain barrier (Fig. 10c).^85^ In this system, the peptide is employed as a transport and targeting element, while the cage scaffold preserves its coordination geometry and encapsulation properties. This selective physiological barrier prevents over 98% of potential neurotherapeutics from reaching target sites. Cage 145 conjugated with 149 demonstrated superior translocation efficiency across a blood–brain barrier model compared to the un-conjugated cage.

The peptide-functionalised system achieved rapid brain accumulation (*t*_1/2_ ≈ 5 minutes), reaching 0.42 ± 0.06% injected dose per gram tissue in CD1 mice when encapsulating [^99m^TcO_4_]^−^, comparable to the free PepH3 peptide performance (0.31 ± 0.07% ID per g). The biodistribution profile demonstrated selectivity, with preferential brain accumulation over peripheral organs. Minimal release of free pertechnetate was evidenced by comparable activity levels in blood, stomach, and thyroid, contrasting with typical accumulation patterns for free [^99m^TcO_4_]^−^. These results indicate that peptide conjugation enables blood–brain barrier traversal without compromising host–guest stability *in vivo*.

### 4.2 Biomolecular recognition and diagnostic imaging

The design of pseudopeptidic molecular cages has evolved into sophisticated stereoselective receptors capable of recognizing biologically-relevant peptide sequences. Here, peptide-derived functionality is exploited to introduce complementary interaction sites within preorganised cage cavities. The imine-based cages developed by Alfonso's group showed good association constants towards Cbz–Ala–Phe–OH as compared to macrocyclic hosts.^101^ ESI-MS screening revealed that structural preorganisation was crucial for efficient peptide recognition, with a mechanism involving aromatic interactions between phenylalanine residues and the cage cavity, complemented by hydrogen bonding between carboxylate groups and amide moieties. These studies identified clear preferences for dipeptides bearing aromatic residues at the C-terminus. The cage obtained from precursor 110 (Fig. 8a) emerged as the most efficient receptor, with binding constants reaching 417 M^−1^ in competitive CD_3_CN/CD_3_OH media, highlighting the importance of cooperative noncovalent interactions, attributed to additional hydrogen bonding capabilities of its serine hydroxyl groups.

Subsequent research focused on the biologically relevant Ac–Glu–Tyr–OH dipeptide, a target sequence for tyrosine kinases.^102^ These enzymes catalyse protein phosphorylation reactions regulating signal transduction, cellular metabolism, and cell cycle progression, with aberrant activity implicated in cancer and metabolic diseases. A remarkable breakthrough was realised through the demonstration of consistent stereoselective recognition across all four stereoisomers of Ac–Glu–Tyr–OH.^102^ Cages obtained from 110 and 111 exhibited selectivity following LL > DD ≥ LD > DL, with the naturally occurring LL stereoisomer binding with highest affinity (631 ± 45 M^−1^ for 110 in aqueous acetonitrile). This behaviour underscores how cage preorganisation and peptide functional group complementarity together enable stereochemical discrimination, representing a rare example of synthetic receptors achieving stereoselective peptide recognition in competitive media, arising from configurationally dependent cooperative interactions between glutamic acid and tyrosine residues with the chiral cage framework.

This stereoselectivity was retained in gas-phase studies using ESI-MS with enantiomer-labelled methods and collision-induced dissociation experiments, suggesting that polar interactions, rather than hydrophobic forces, primarily govern selectivity. Spectroscopic studies and molecular modelling revealed a three-site binding mechanism involving carboxylate coordination, aromatic encapsulation through stacking, and acetyl group stabilisation. In this context, peptide side-chain functionality plays a decisive role in defining binding mode and selectivity, as the phenolic hydroxyl group of tyrosine provides additional hydrogen bonding, explaining enhanced binding compared to phenylalanine analogues.

Beyond peptide recognition within discrete host cavities, peptide-derived stereochemical information has also been exploited to programme the selective recognition of higher-order nucleic acid architectures, demonstrating how preorganisation and chirality encoded in peptide frameworks can be translated into macromolecular targeting.

In a conceptually distinct yet philosophically related strategy, Vázquez and co-workers demonstrated that peptide-derived ligands can encode stereoselective recognition of noncanonical DNA structures through metallosupramolecular helicates. Oligocationic peptide ligands incorporating heterochiral β-turn sequences self-assemble with Fe^II^ or Co^II^ ions into chiral dinuclear helicates that bind three-way DNA junctions with sub-micromolar affinity.^54^ The stereochemical information embedded in the peptide sequence directs the formation of ΛΛ or ΔΔ helicates, whose trigonal geometry and shape complementarity enable insertion into the hydrophobic cavity at the DNA junction branch point, assisted by electrostatic interactions with the phosphate backbone. Rhodamine-labelled FeII helicates were shown to internalise into living cells and selectively localise at DNA replication foci, providing the first designed fluorescent probes capable of visualising three-way DNA junctions *in cellulo*. Building on this platform, the same group subsequently introduced redox-active Cu^II^ peptide helicates, yielding the first chemical nuclease displaying selective cleavage of three-way junctions both *in vitro* and in mammalian cells.^53^ Mechanistic studies implicated a reactive oxygen species mediated pathway consistent with metal-centred superoxide formation, while TUNEL assays in synchronised cells confirmed selective DNA damage at replication foci. Collectively, these studies highlight how peptide-encoded stereochemistry, preorganisation, and metal coordination can be combined to achieve selective recognition and reactivity towards transient biological DNA architectures.

### 4.3 Molecular separation and environmental remediation

Suitably designed peptides can display the useful ability to self-assemble into gelling nanostructures, with sequences as short as 2–3 amino acids peptides creating robust hydrogels through hierarchical assembly processes.^114^ These minimalistic gelators form fibrillar networks that trap large solvent volumes, creating soft materials with tuneable mechanical properties and stimuli-responsive behaviours.^115^ Gelation is driven by non-covalent interactions including hydrogen bonding, aromatic stacking, and electrostatic interactions, modulated by pH, temperature, and ionic strength.^116^ Peptide gels show compatibility with metal ions, where metal coordination influences gel formation, stability, and mechanical properties.^117–119^

**Fig. 11:**
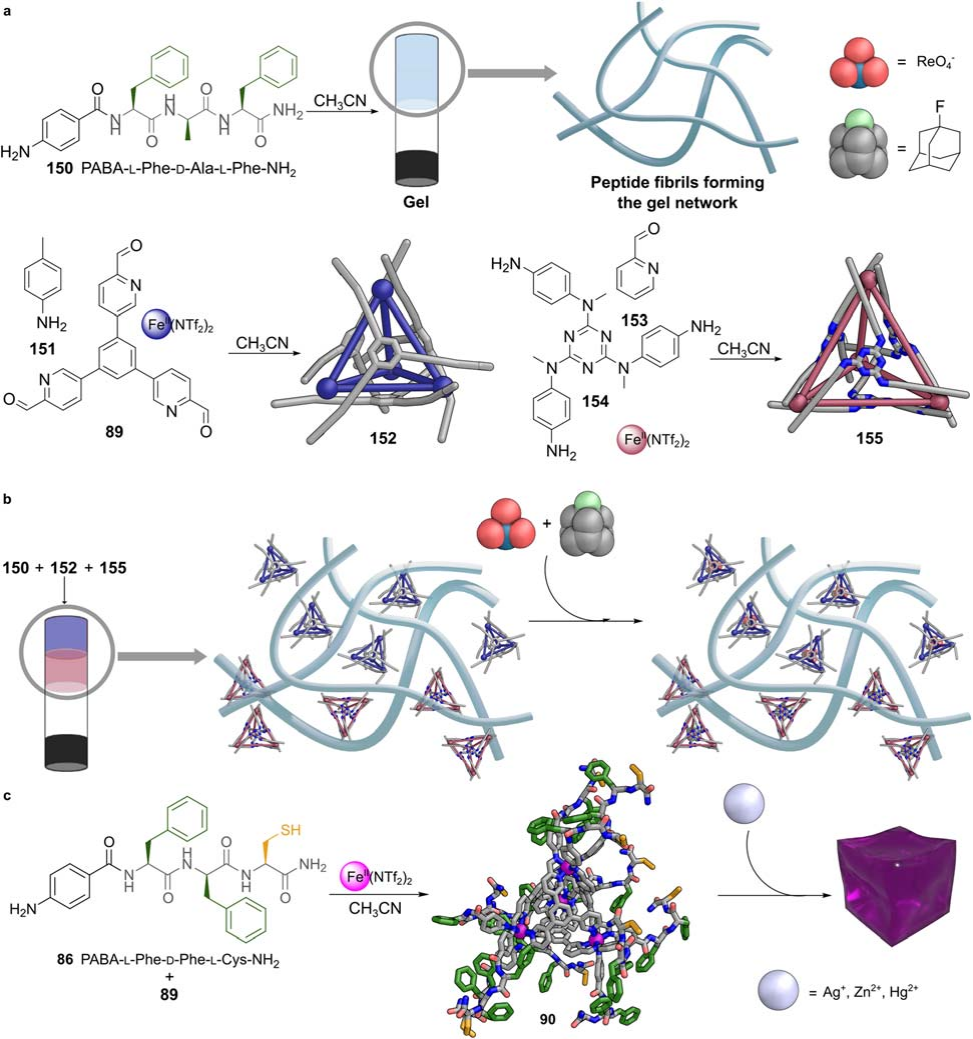
Schematic illustration of the dual supramolecular system combining a self-assembling tripeptide hydrogelator and two discrete tetrahedral FeII4L4 cages. (a) The tripeptide PABA–l-Phe–d-Ala–l-Phe–NH_2_ (150) undergoes hierarchical self-assembly into fibrillar networks that entrap solvent molecules, forming a viscoelastic gel. Within this matrix, two chemically distinct MOCs (152 and 155) are incorporated without disrupting gelation. (b) A layered peptide gel spatially confines the cages while preserving their intrinsic molecular recognition properties. Each cage exhibits orthogonal guest selectivity in acetonitrile, binding ReO_4_^−^ (for 152) and fluoroadamantane (for 155), respectively. (c) Cage 90 undergoes gelation through coordination of the thiols of the Cys to a second external metal ion, *i.e.*, Ag^+^, Zn^2+^, or Hg^2+^ (white spheres). The gel matrix forms through a two-phase kinetic process, producing spherical nuclei whose size and mechanical properties depend on the metal ion. Colour code for rendered structures: peptide backbone and aromatic scaffold = grey, N = blue, O = red, S = yellow, Fe = magenta, Ag, Zn, and Hg = light grey, F = light green.

The combination of peptide gels with metal–organic cages creates hierarchically organised, dual-porosity soft materials.^120^ As shown in Fig. 11a, embedment of [Fe^II^_4_L_4_]^8+^ cages within supramolecular tripeptide gels enables spatial separation of different molecular guests through selective encapsulation during diffusion. This hierarchical assembly exploits both mesoscopic gel network pores and cage cavities for selective guest entrapment, offering distinct pore sizes and physico-chemical properties for controlled molecular transport and separation.^121^ An additional advantage is the increased cage stability against acid-mediated hydrolysis in the presence of the peptide gel matrix.

Recent developments produced cages 90–92 (Fig. 11b) whereby typical aniline peripheral ligands^122^ have been substituted with PABA–l-Phe–d-Cys–l-Phe–NH_2_, PABA–l-Phe–d-Phe–l-Cys–NH_2_, or PABA–l-Phe–d-Met–l-Phe–NH_2_ that incorporate peripheral cysteine or methionine residues. In these systems, peptide sequence and residue positioning directly influence supramolecular behaviour. Interestingly, gelation was attained only for cage 91 upon exposure to Ag^+^, Zn^2+^, or Hg^2+^ ions through thiol-metal coordination.^70^ A likely explanation is the limiting steric hindrance of the coordinating Cys/Met in cages 90 and 92, which feature such amino acids in the central position of the peptide sequence. Rheological characterisation of the gels obtained from cage 91 revealed variable gel stiffness from 1.6 kPa (Ag^+^) to 3.9 kPa (Hg^2+^), reflecting different metal–sulfur coordination affinities. The gelation process exhibits distinctive two-stage kinetics: an initial lag phase followed by rapid matrix formation. Silver ions display the longest nucleation phase, forming larger spherical nuclei, while zinc and mercury promote faster gelation with smaller nuclei. A direct morphology–kinetics correlation is thus established, where extended lag phases favour the formation of larger nuclei and stronger coordination accelerates network development. The resulting materials exhibit spherical nuclei interconnected within the gel matrix, contrasting with typically fibrillar networks arising from the self-assembly upon oxidation of similar peptide sequences in the absence of cages.^123^ The same oxidative approach to crosslink the cysteine residues of cages 90 or 91 through disulfide bridges did not lead to gelation, further highlighting the constraints in their supramolecular behaviour when self-assembling tripeptide motifs are bound at the cage vertices.^70^ Nevertheless, the metal-triggered gelation may enable promising environmental applications, particularly heavy metal remediation, through selective coordination to peripheral cysteine residues.

Environmental remediation represents an arena in which peptide cages excel. Beyond metal ion capture, they bear the potential to address critical water contaminants, such as hydrocarbons. Cage 106 (Fig. 11c) demonstrates remarkable selectivity for NaF in aqueous environments, with binding affinities of 3.08 × 10^3^ M^−1^, achieved through an atypical entropy-driven endothermic mechanism.^72^ This anti-Hofmeister behaviour distinguishes these systems from conventional anion-binding receptors. The cage structural flexibility accommodates highly hydrated fluoride ions without requiring significant desolvation, while maintaining selectivity over other halides. SCXRD analysis revealed highly hydrated binding pockets where water molecules participate in hydrogen bonding networks with encapsulated fluoride anions.

A variant of cage 106 with ligand 103 substituted with a cyclohexyl group demonstrated exceptional efficacy in removing per- and polyfluoroalkyl substances (PFAS) from contaminated water. Batch equilibrium experiments showed 70–80% removal of perfluorooctanoic acid (PFOA) within ten minutes at 1 µg L^−1^, reaching completion within six hours. At higher concentrations (50 µg L^−1^), the system achieved 95% removal efficiency within four hours. A Langmuir isotherm model yielded an affinity coefficient of 2.8 × 10^5^ M^−1^ and maximum capacity of 19.99 mg g^−1^. The cage maintained selectivity for fluorinated compounds over non-fluorinated analogues, with PFOA removal exceeding 97%, while octanoic acid removal remained at 20% under identical conditions. The system demonstrated resilience in natural water, maintaining effective PFAS capture despite the presence of organic matter.

Noteworthily, the applications described throughout this section arise exclusively from hybrid peptide-aromatic cages. Such cages derive their functionality from rigid aromatic cores that provide structural stability and preorganisation, while peptide components are incorporated to introduce chemical functionality, responsiveness, and selectivity. This division of roles enables molecular recognition across chemically diverse targets, ranging from therapeutic agents to environmental pollutants. Extension of such recognition to polar biomolecules in aqueous media represents the next step. These cages create multivalent interfaces, where peptide units generate selectivity for biological targets such as integrin receptors, although broader validation across different receptor classes remains limited. Furthermore, heterochirality may generate asymmetric binding surfaces for greater target discrimination. Environmental responsiveness enables pH-triggered ion transport and metal-induced gelation, demonstrating how peptide components confer adaptive behaviour. Incorporation of enzymatic triggers or redox-sensitive elements would expand this functional repertoire. Peptide-metal coordination produces materials with tuneable mechanical properties for biomedical applications, where reversible crosslinking strategies might yield self-healing characteristics. By contrast, the purely peptidic cages from Section 2 lack these features. Most of these assemblies employ homochiral sequences with limited side-chain diversity, reflecting a focus on geometric validation rather than functional design. This emphasis on structure has naturally preceded the emergence of application-oriented studies. The design principles underlying hybrid cage functionality illustrate a possible pathway towards purely peptidic architectures to transition from proof-of-concept structures to functional platforms capable of molecular recognition, targeted delivery, and stimuli-responsive assembly.

## 5. Conclusions and future perspectives

The exploration of peptide-based supramolecular cages has highlighted two distinct yet overlapping and complementary structural design strategies, each with unique advantages and inherent limitations. Purely peptidic cages, constructed entirely from peptide sequences, capitalise on intrinsic conformational preferences and metal coordination to form intricate topological motifs, such as knots, catenanes, and interlocked structures. These architectures exhibit a high level of complexity and topological precision, rivalling natural protein assemblies. Despite their sophisticated designs, purely peptidic cages encounter challenges that can include limited aqueous stability and stringent assembly conditions, typically requiring organic solvents or precisely controlled environments. Consequently, their applications so far remain linked to structural elucidation rather than functional exploitation.

In contrast, hybrid peptide-aromatic cage systems combine rigid aromatic frameworks with peptide functionalities, offering structural robustness and modularity. This approach enables the systematic fine-tuning of properties through targeted amino acid modifications, broadening the practical applicability of such cages. These hybrid systems have found uses in areas such as drug delivery, selective molecular recognition, and environmental remediation. However, they are not without drawbacks. Their synthetic complexity and the potential metabolic instability of their aromatic scaffolds, combined with challenges in scalability, may present obstacles to implementation.

Several key design principles emerge from an analysis of both approaches, although the field remains at an early stage and the extraction of general, predictive rules is still challenging. The structure–function relationships that we elucidate herein should be thus interpreted as emerging trends rather than universally established design principles. The careful incorporation of diverse amino acid side chains and the strategic use of heterochirality have enabled selective molecular recognition and stereochemical control of cage cavities in specific systems. Additionally, the development of stimulus-responsive features, including pH-sensitive behaviours, metal-triggered structural rearrangements, and adaptive guest binding, has enriched the functional landscape of peptide-based cages. These properties underscore the inherent complementarity between purely peptidic and hybrid cage design strategies, suggesting that future advances will likely integrate the strengths of both approaches.

A promising avenue thus involves merging the versatility of peptide backbones with the geometric precision provided by preorganised scaffolds. This hybrid strategy may reduce entropic penalties associated with self-assembly, while preserving the functional diversity inherent in peptides. Introducing non-natural building blocks, such as β-amino acids or peptoids, provides a robust approach to impose conformational constraints at the monomeric level.^124–128^ These residues may stabilise ordered structures without compromising biocompatibility, although their broader impact on assembly and function remains to be systematically assessed. Selecting these components involves carefully balancing structural rigidity, aqueous solubility, and potential immunogenicity to ensure optimal performance in biological contexts.

The introduction or alteration of stereogenic centres allows the exploration of a broader range of supramolecular architectures. Moreover, stereochemical differentiation provides an effective strategy to independently modulate backbone geometry and functional group orientation, thereby enhancing structural control and specificity.^18^

By analysing natural peptide sequences and microdomains, researchers can isolate critical minimal motifs that promote targeted folding or molecular recognition. Incorporating these minimalist motifs into larger synthetic architectures streamlines the design process and leverages functionalities optimised through evolutionary selection.^129–131^

Advances in modular functionalisation using bioorthogonal chemistries have the potential to further enhance the versatility of peptide-based cages. Click-chemistry reactions, inverse-electron-demand Diels–Alder cycloadditions, and other bioorthogonal methods facilitate rapid and selective conjugation of diagnostic probes, therapeutic agents, or catalytic sites.^132–134^ Such late-stage functionalisation allows iterative optimisation without necessitating complete resynthesis of the peptide scaffold.

Finally, given the current youth and heterogeneity of the field, computational approaches such as molecular dynamics simulations and data-driven methods including machine learning should be regarded as long-term opportunities rather than immediate solutions. At present, the limited availability of consistent experimental datasets and validated computational models restricts their direct application to peptide-based cages. Nevertheless, conceptual guidance may be drawn from related areas, particularly protein folding and protein cage assemblies, where extensive experimental and computational studies have enabled the identification of stabilising motifs and assembly pathways.^135–137^ As the field matures and more systematic structure–property data become available, these approaches may support hypothesis-driven design and prioritisation of candidate sequences, complementing chemically informed strategies rather than replacing them.^138,139^ Together, these strategies promise to propel peptide-based supramolecular cages beyond current constraints, enabling new opportunities for practical applications.

## Author contributions

SA: writing – original draft, visualisation. HX: writing – original draft, visualisation. JRN: conceptualisation, project administration, writing – review and editing. SM: conceptualisation, project administration, writing – review and editing.

## Conflicts of interest

There are no conflicts to declare.

## Data Availability

No primary research results, software, or code have been included, and no new data were generated or analysed as part of this review.
